# Supplementary material for: Facile Synthesis of 2H-Benzo[h]Chromenes via an Arylamine-Catalyzed Mannich Cyclization Cascade Reaction
Source: Molecules. 2021 Jun 12;26(12):3617. doi: 10.3390/molecules26123617 (PMC8231631; doi:10.3390/molecules26123617)
Supplement: Supplementary file 1 [file molecules-26-03617-s001.zip › molecules-1202113-supplementary.pdf]

Supplementary Materials

Facile Synthesis of 2*H*-Benzo[*h*]chromenes via an Arylamine-catalyzed Mannich-cyclization Cascade Reaction

Yueteng Zhang[1,2]*, Peng Ji[1], Xiang Meng[1], Feng Gao[1], Fanxun Zeng[1], Wei Wang[1]*

[1]Departments of Pharmacology and Toxicology and Chemistry and Biochemistry, and BIO5 Institute, University of Arizona, Tucson, Arizona 85721, USA
[2]The School of Basic Medical Sciences, The Academy of Medical Science, Zhengzhou University, 100 Kexue Avenue, Zhengzhou, 450001, Henan, China

Original ^{1}H and ^{13}C NMR spectra

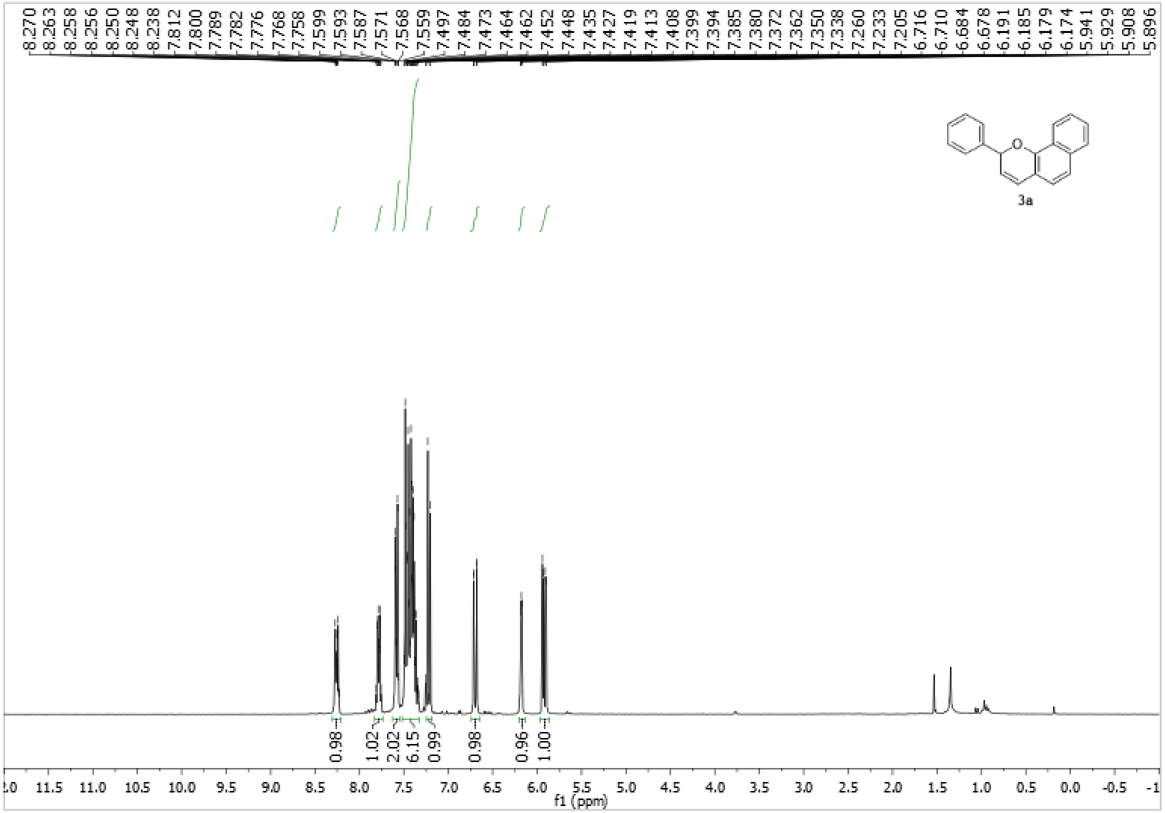

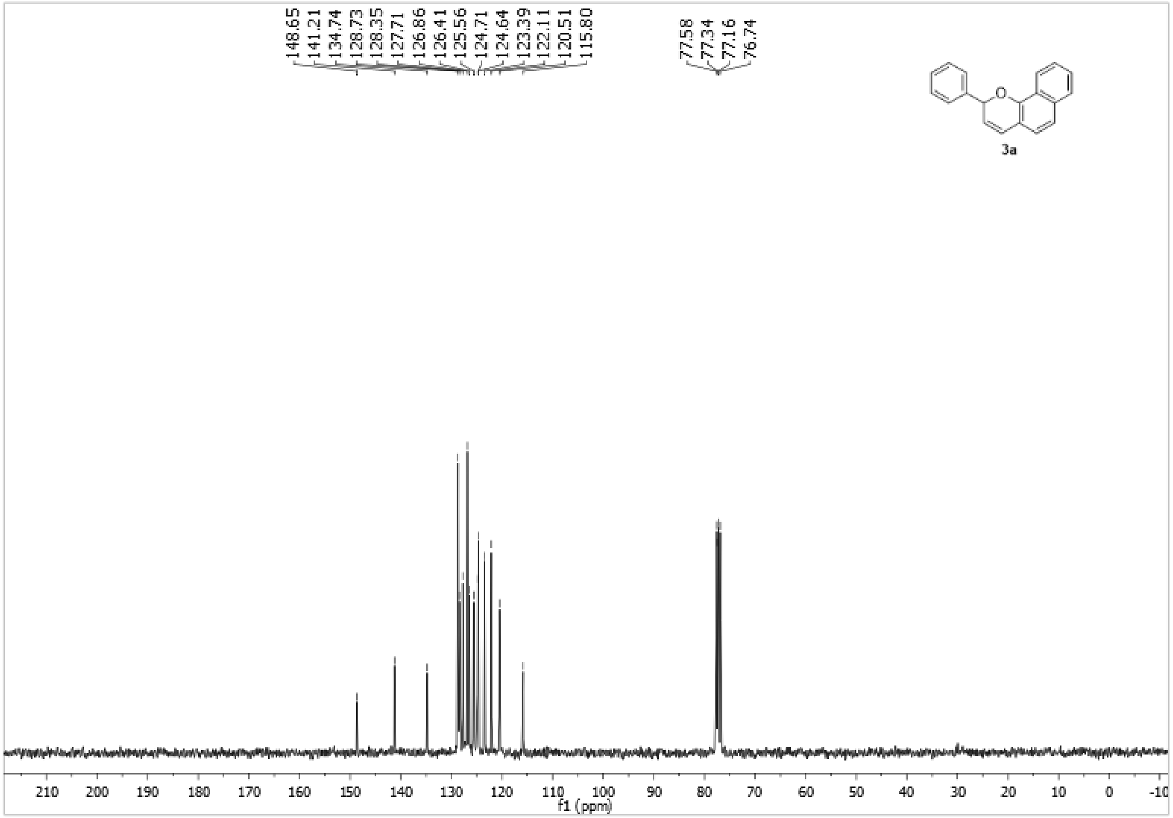

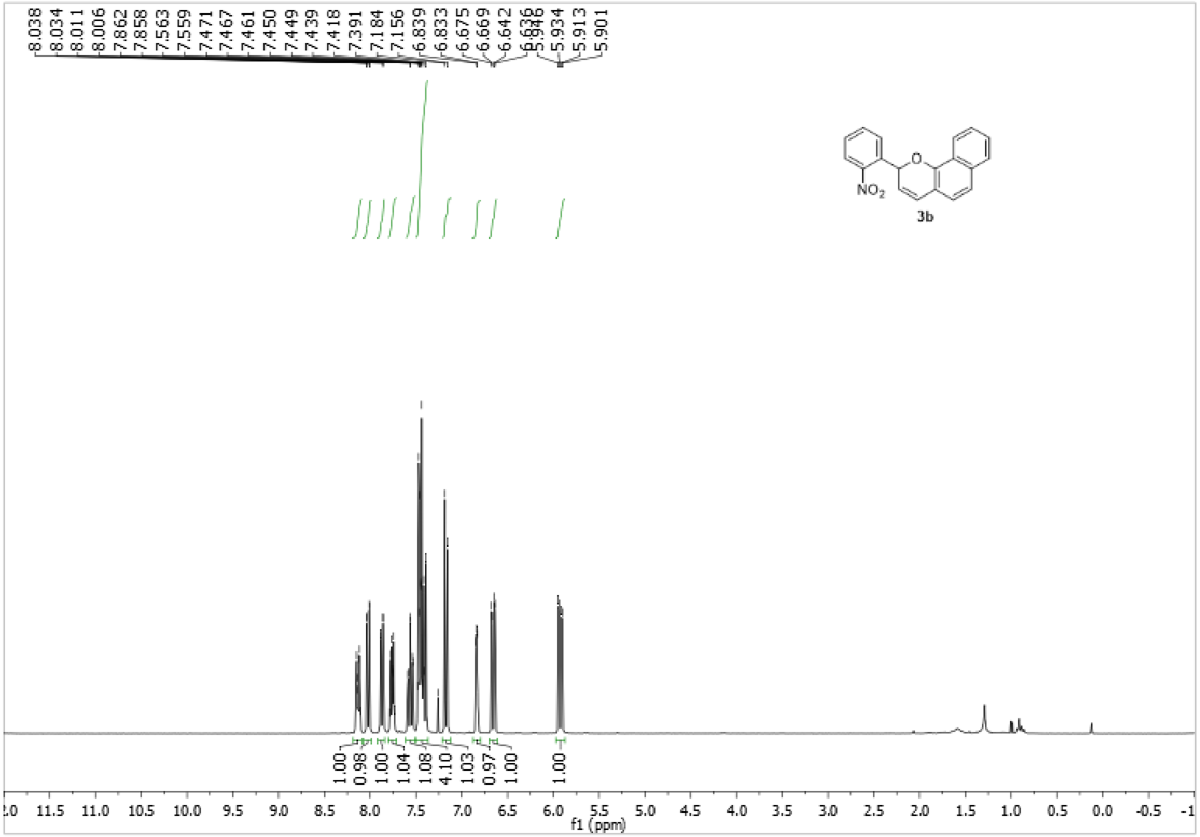

8.038
8.034
8.011
8.006
7.862
7.858
7.563
7.559
7.471
7.467
7.461
7.450
7.449
7.439
7.418
7.391
7.184
7.156
6.839
6.833
6.675
6.669
6.642
5.934
5.913
5.901
NO2
3b
1.00
0.98
1.00
1.04
1.08
4.10
1.03
0.97
1.00
1.00
f1 (ppm)

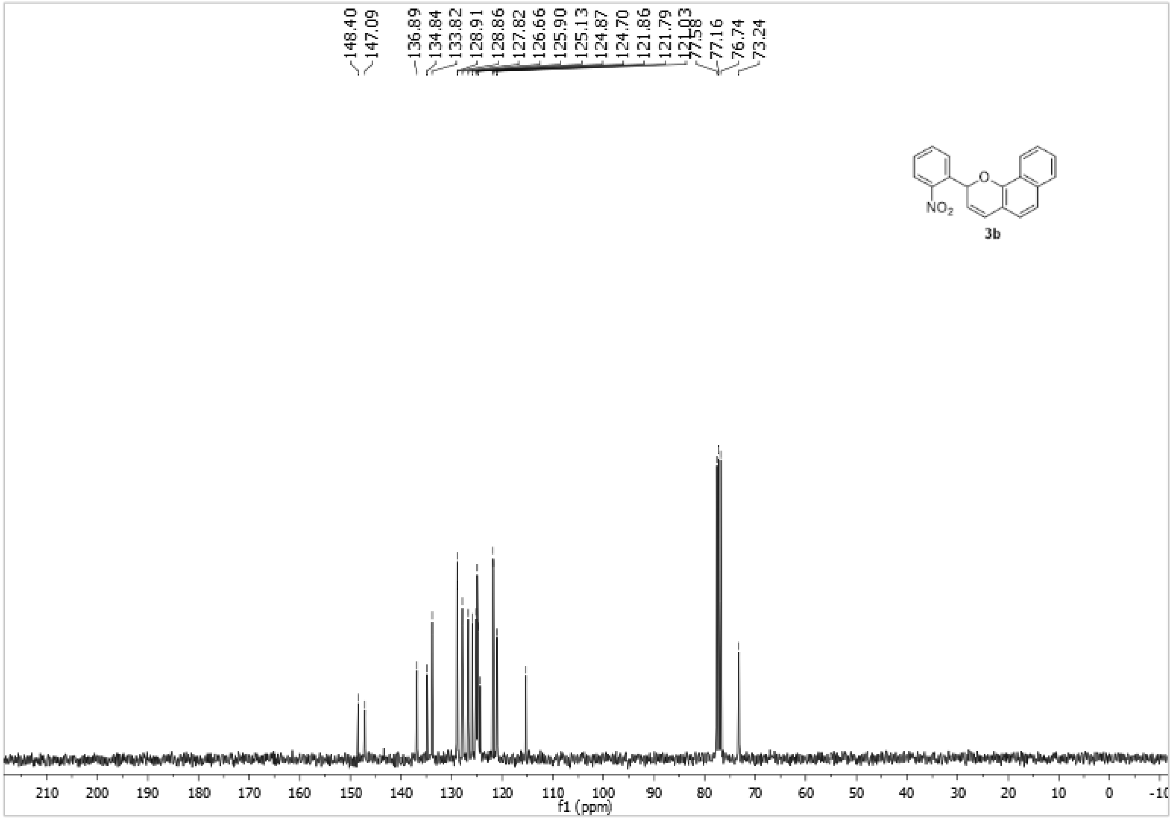

148.40
147.09
136.89
134.84
133.82
128.91
128.86
127.82
126.66
125.90
125.13
124.87
124.70
121.86
121.79
121.03
77.58
77.16
76.74
73.24
NO2
3b
f1 (ppm)

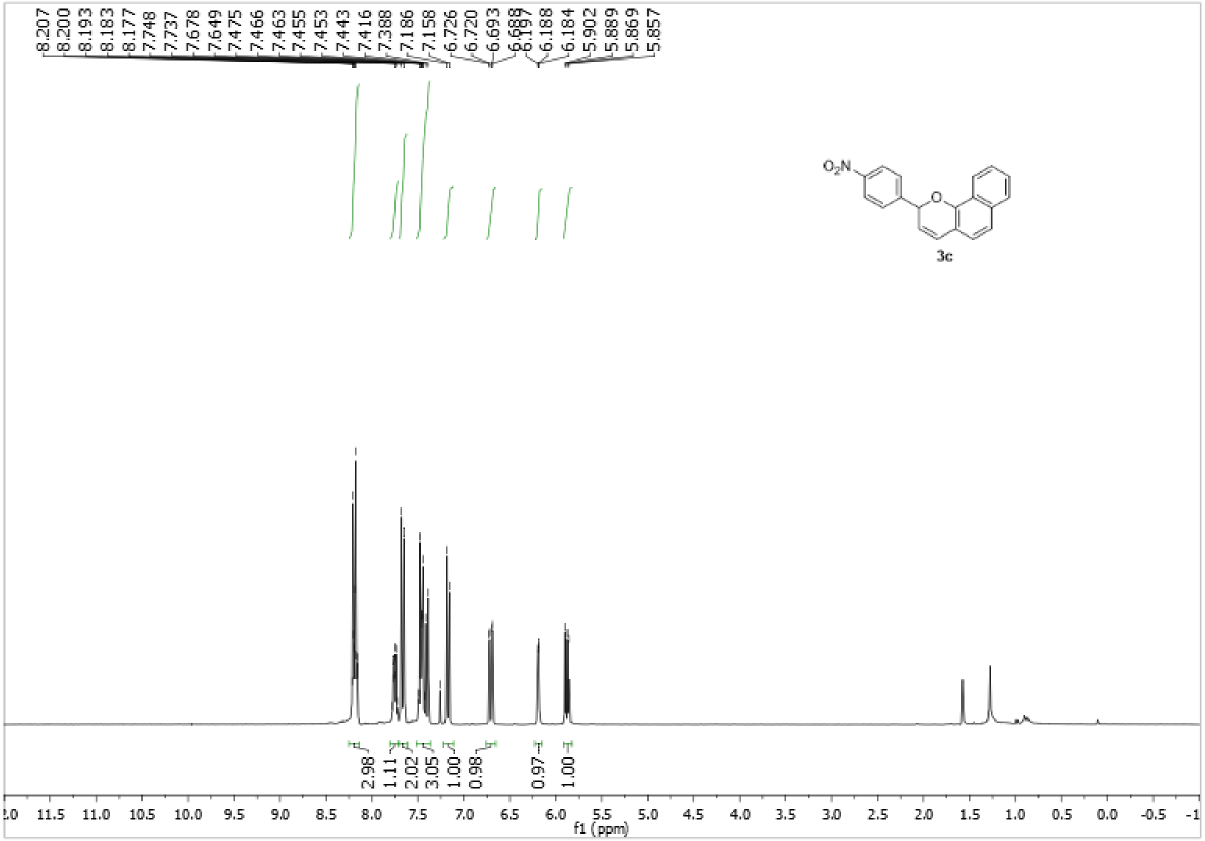

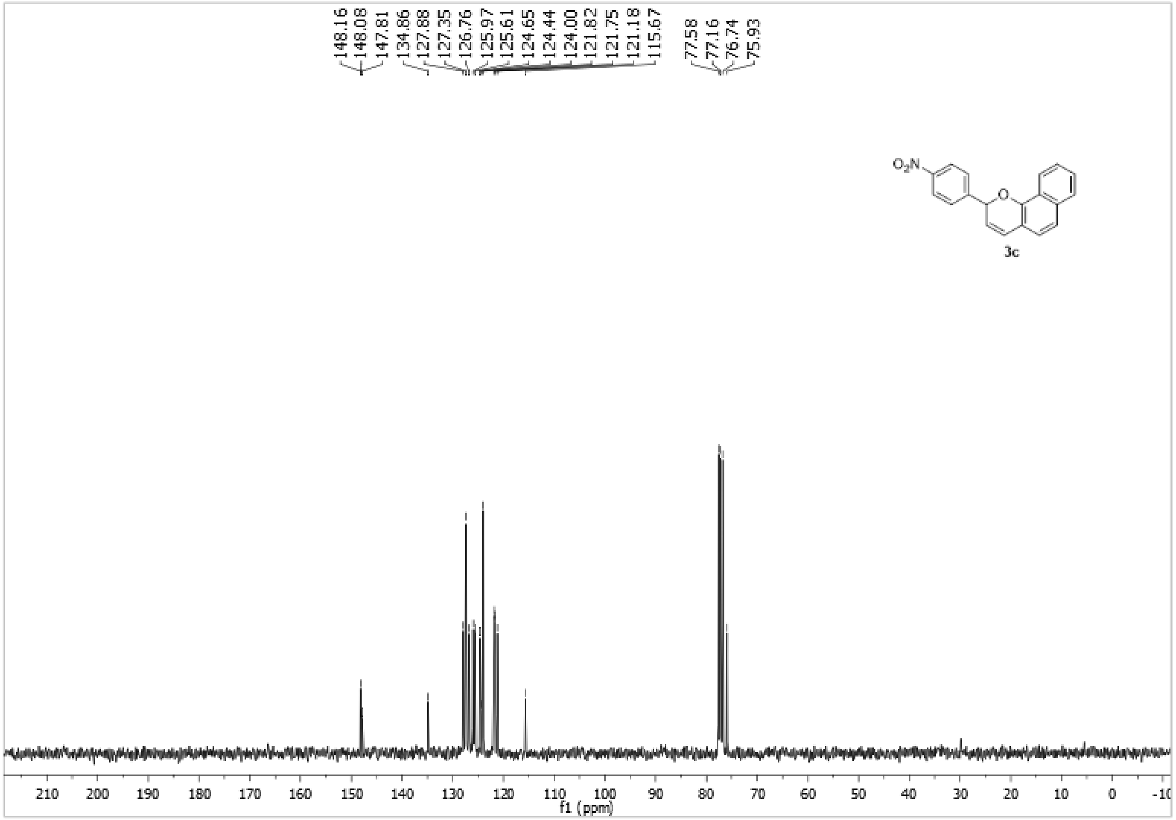

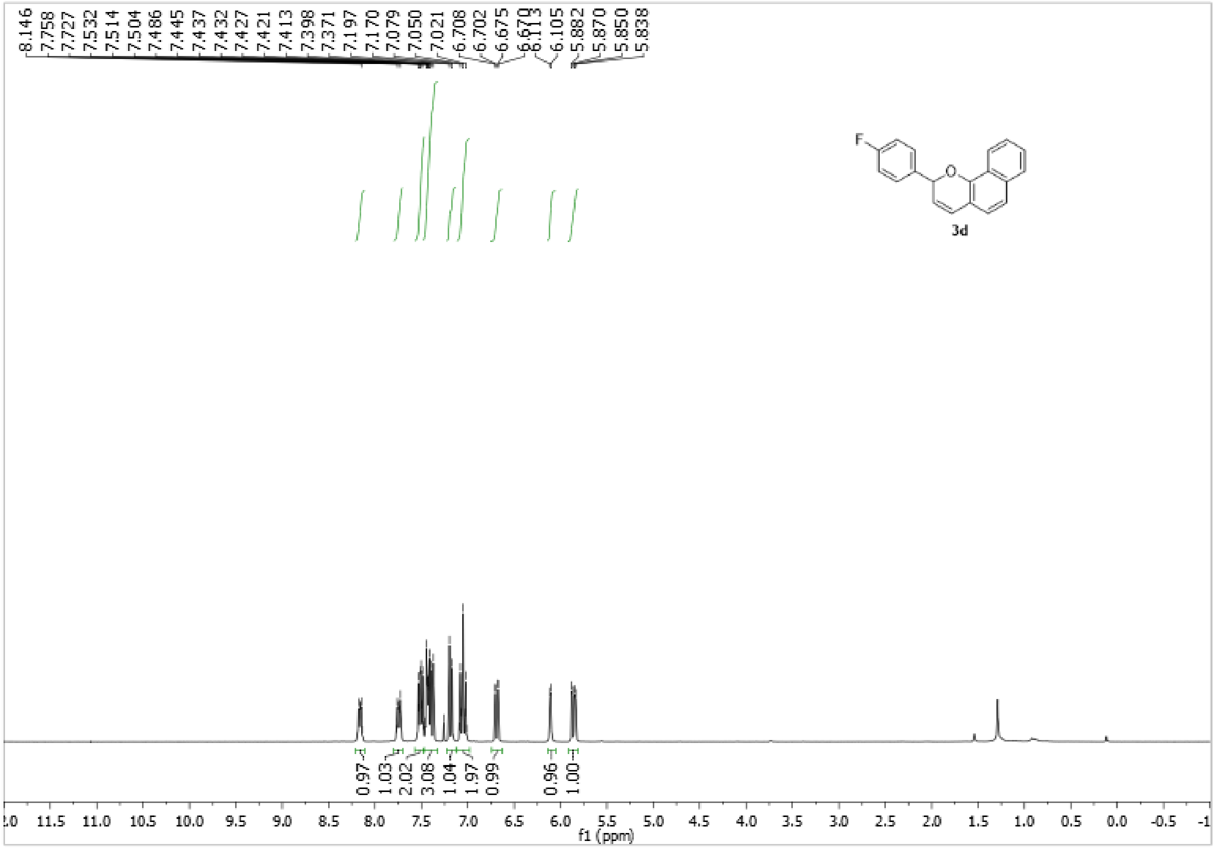
8.146
7.758
7.727
7.532
7.514
7.504
7.486
7.445
7.437
7.432
7.427
7.421
7.413
7.398
7.371
7.197
7.170
7.079
7.050
7.021
6.708
6.702
6.675
6.670
6.113
6.105
5.882
5.870
5.850
5.838
F
O
3d
0.97
1.03
2.02
3.08
1.04
1.97
0.99
0.96
1.00
f1 (ppm)

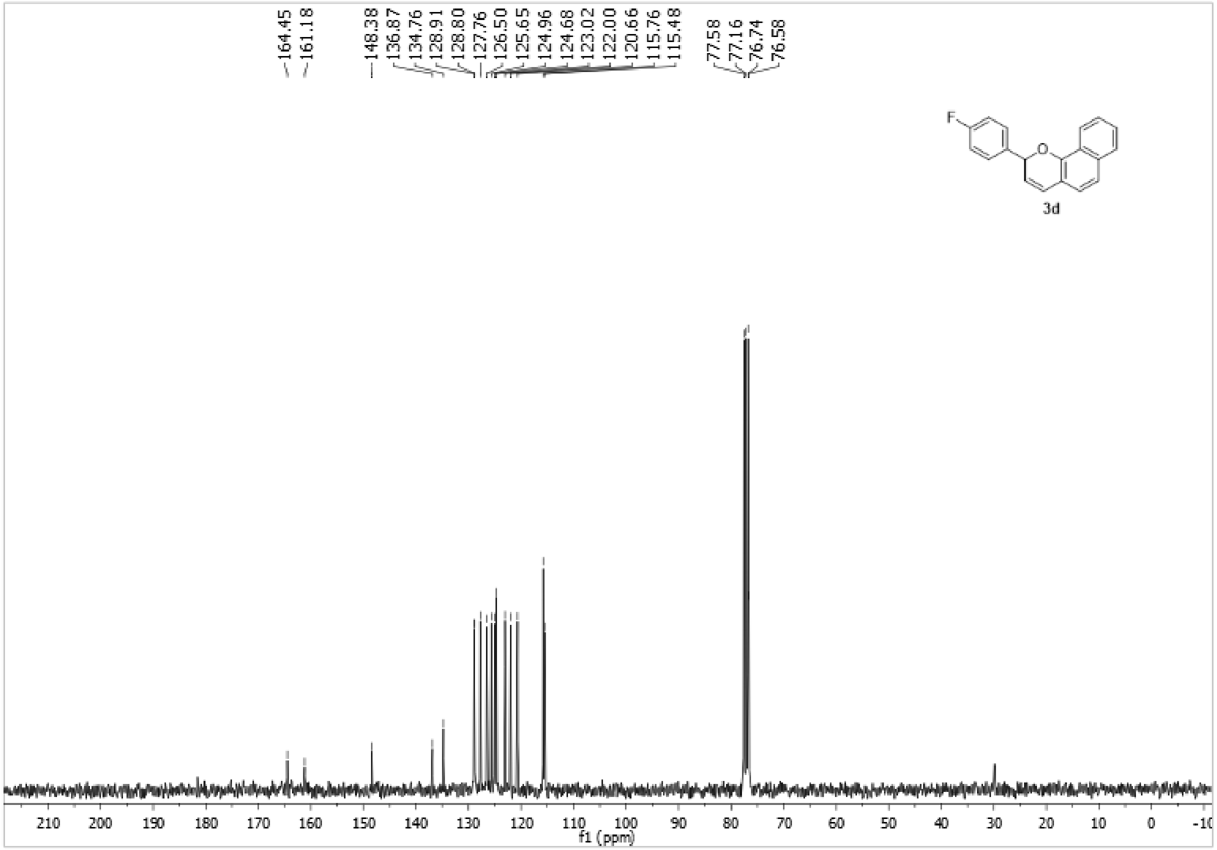
164.45
161.18
148.38
136.87
134.76
128.91
128.80
127.76
126.50
125.65
124.96
124.68
123.02
122.00
120.66
115.76
115.48
77.58
77.16
76.74
76.58
F
O
3d
f1 (ppm)

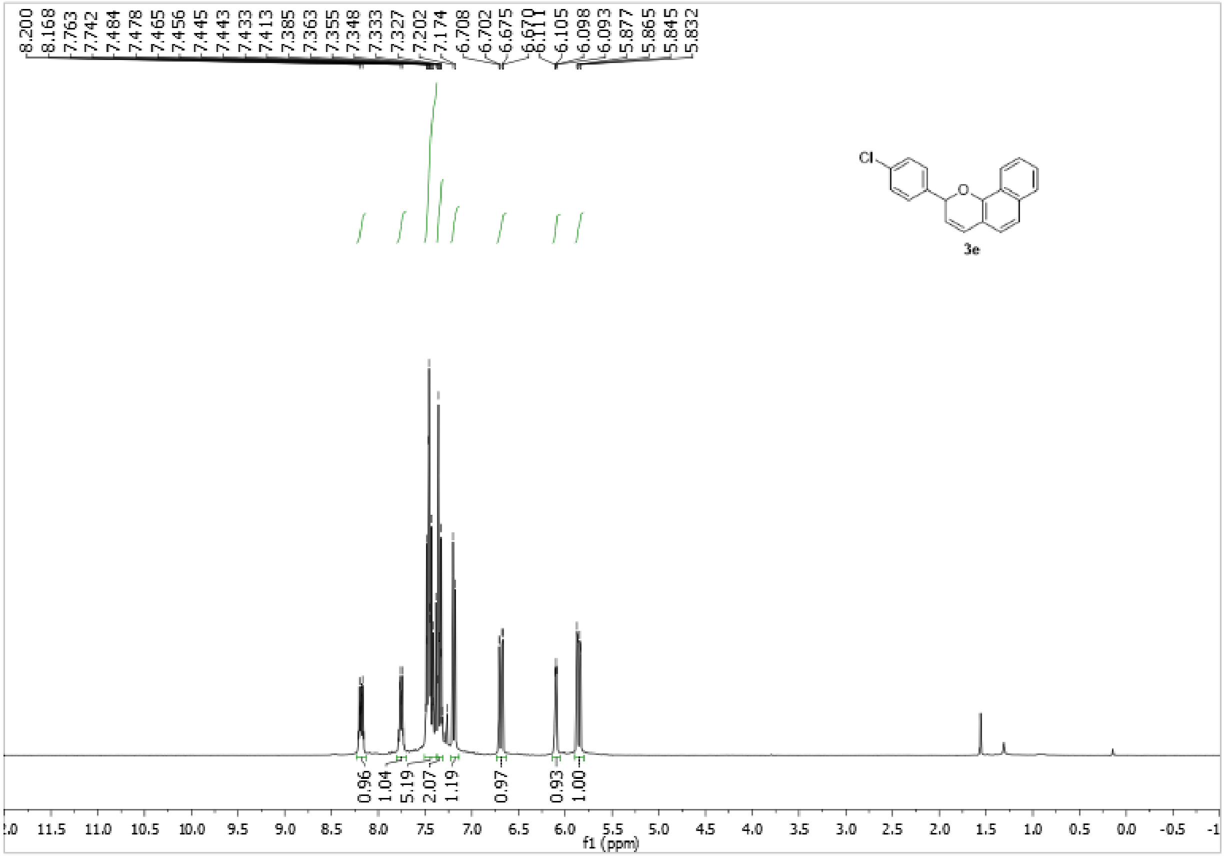
8.200
8.168
7.763
7.742
7.484
7.478
7.465
7.456
7.445
7.443
7.433
7.413
7.385
7.363
7.355
7.348
7.333
7.327
7.202
7.174
6.708
6.702
6.675
6.670
6.111
6.105
6.098
6.093
5.877
5.865
5.845
5.832
Cl
O
3e
0.96
1.04
5.19
2.07
1.19
0.97
0.93
1.00
f1 (ppm)

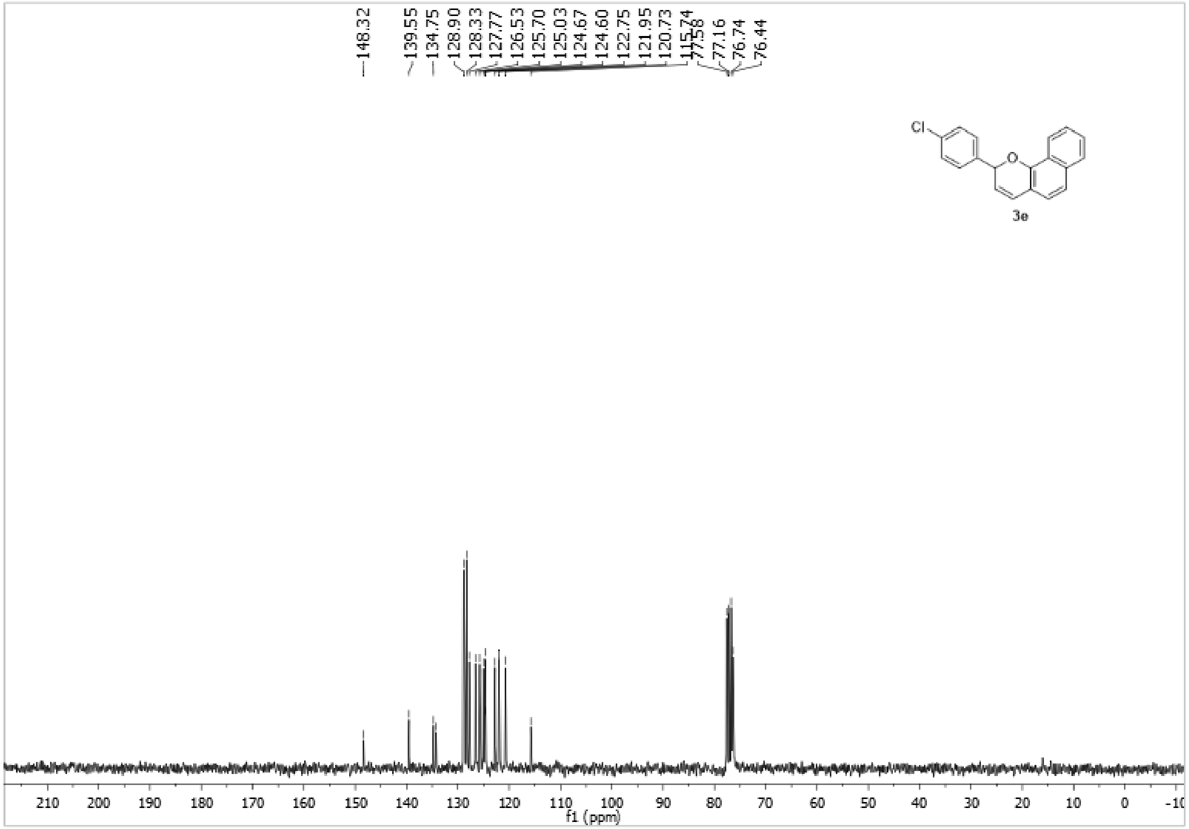
148.32
139.55
134.75
128.90
128.33
127.77
126.53
125.70
125.03
124.67
124.60
122.75
121.95
120.73
115.74
77.58
77.16
76.74
76.44
Cl
O
3e
f1 (ppm)

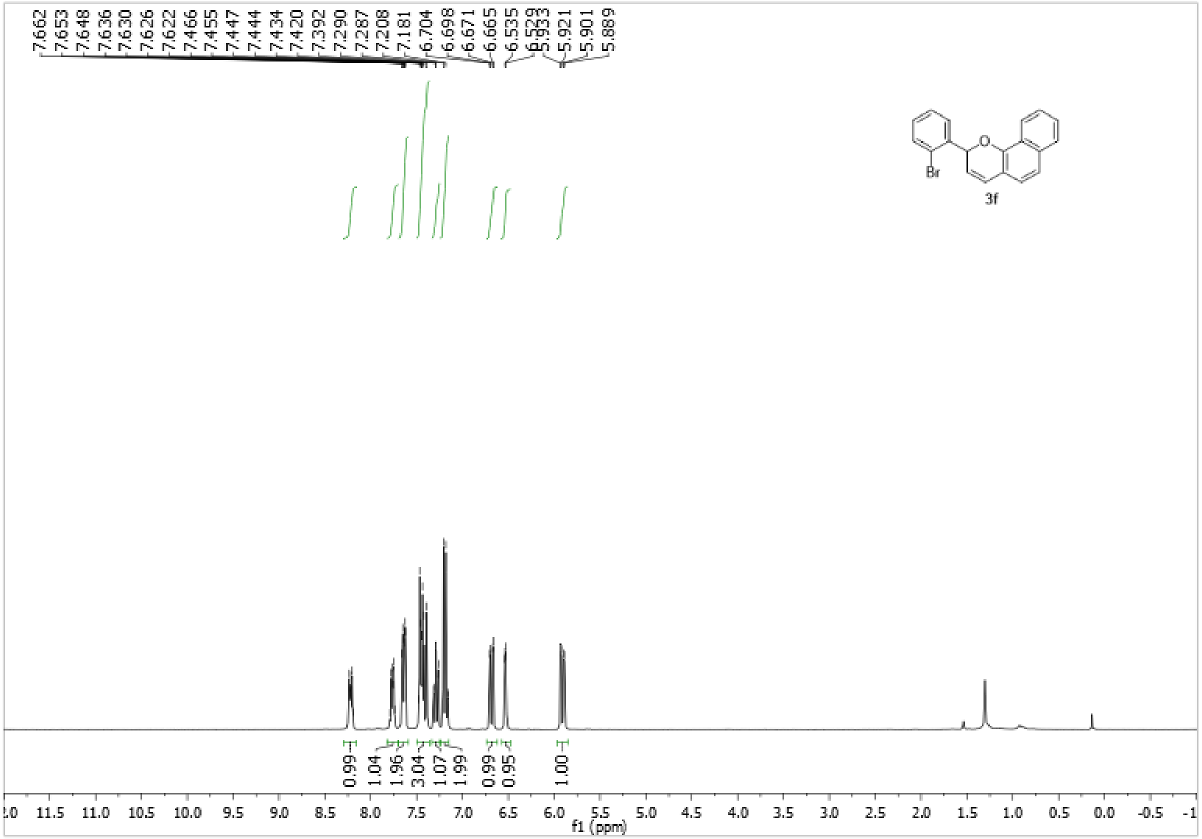

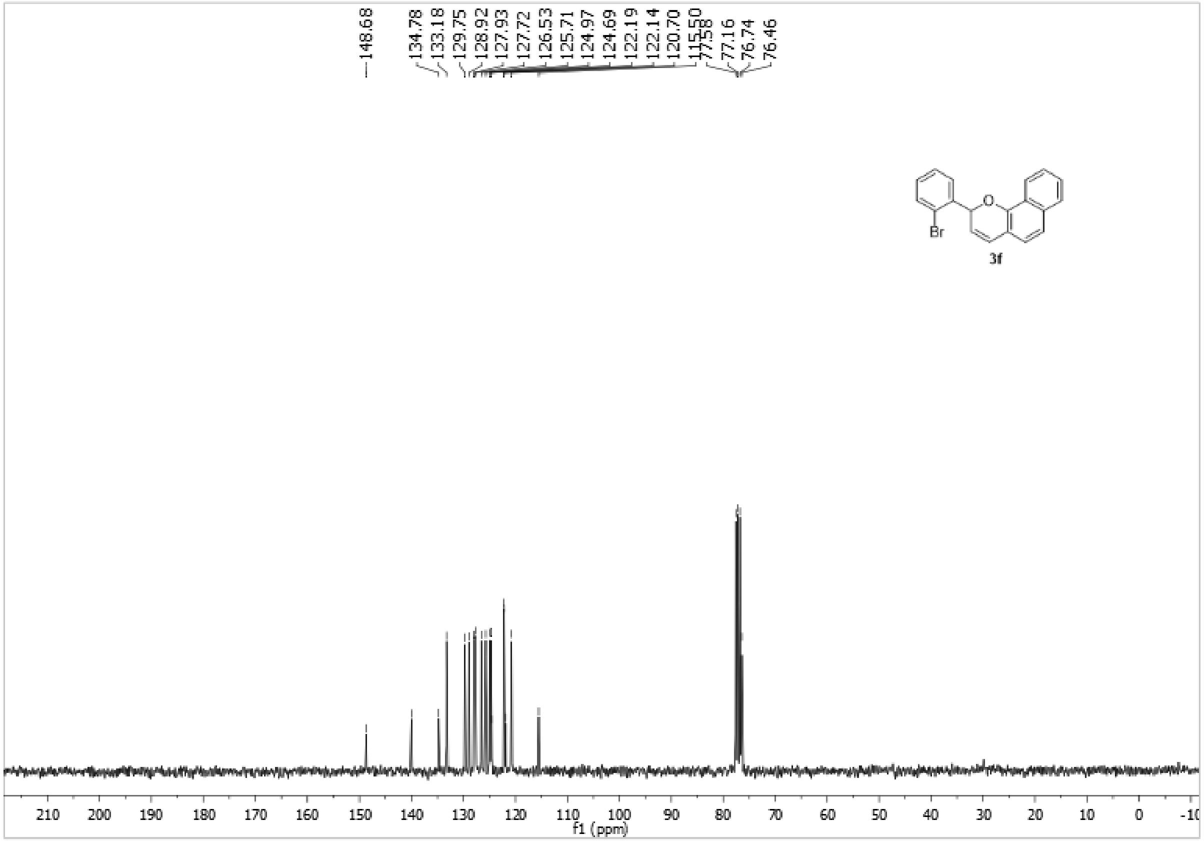

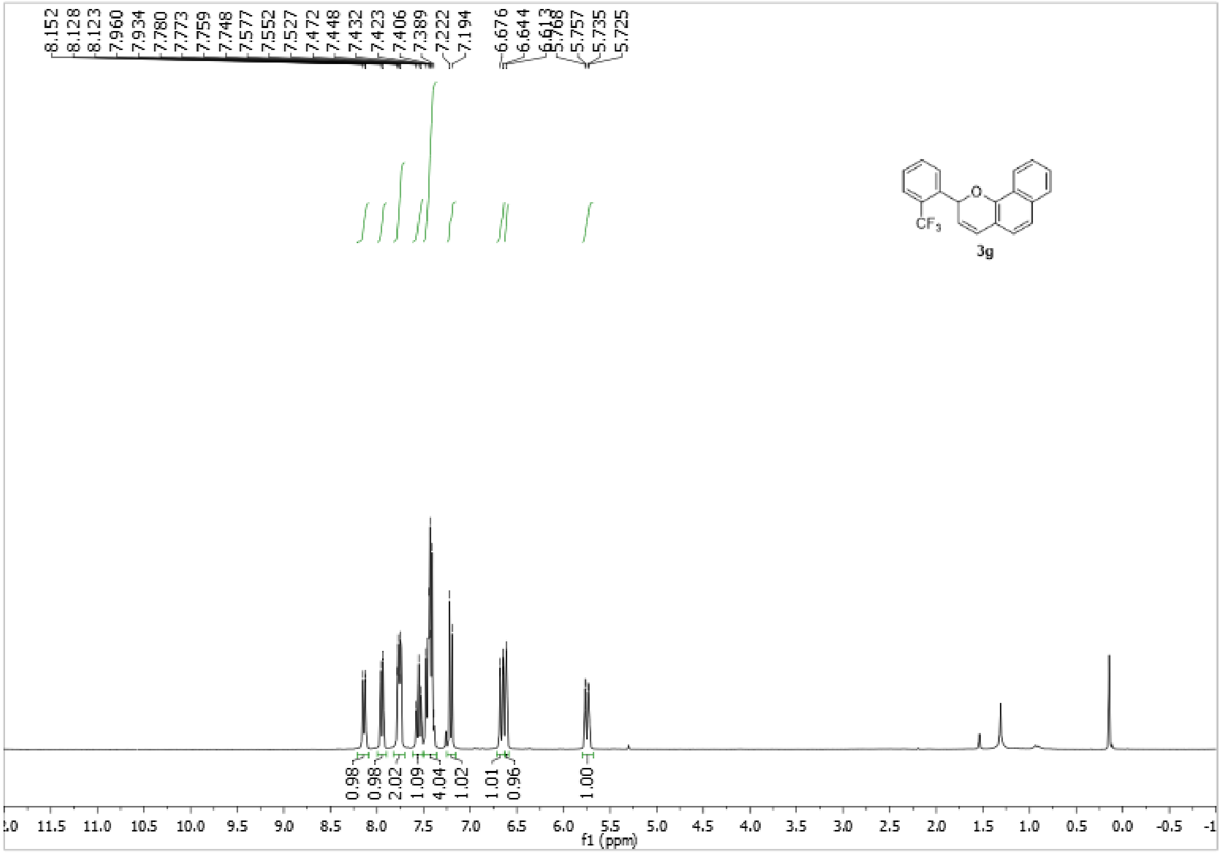
8.152 8.128 8.123 7.960 7.934 7.780 7.773 7.759 7.748 7.577 7.552 7.527 7.472 7.448 7.432 7.423 7.406 7.389 7.222 7.194 6.676 6.644 6.613 5.768 5.757 5.735 5.725
CF3
3g
0.98 0.98 2.02 1.09 4.04 1.02 1.01 0.96 1.00
f1 (ppm)

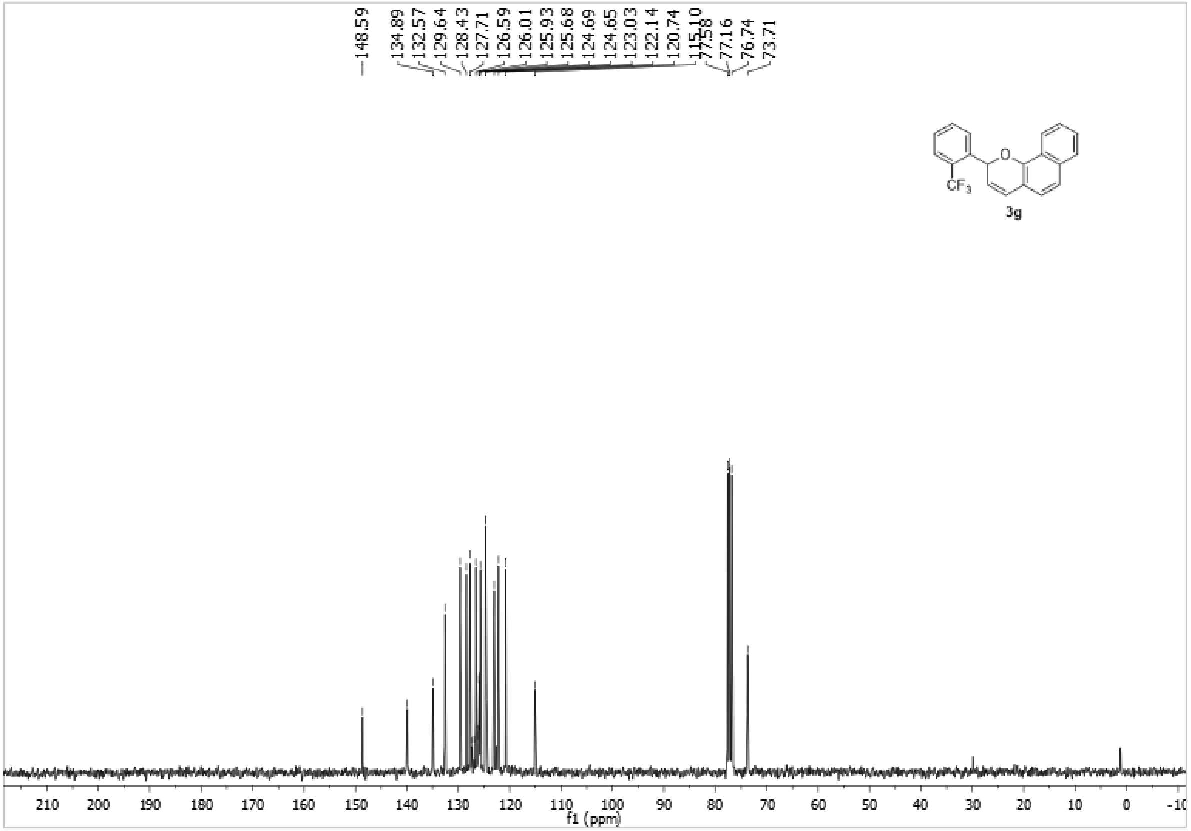
148.59 134.89 132.57 129.64 128.43 127.71 126.59 126.01 125.93 125.68 124.69 124.65 123.03 122.14 120.74 115.10 77.58 77.16 76.74 73.71
CF3
3g
f1 (ppm)

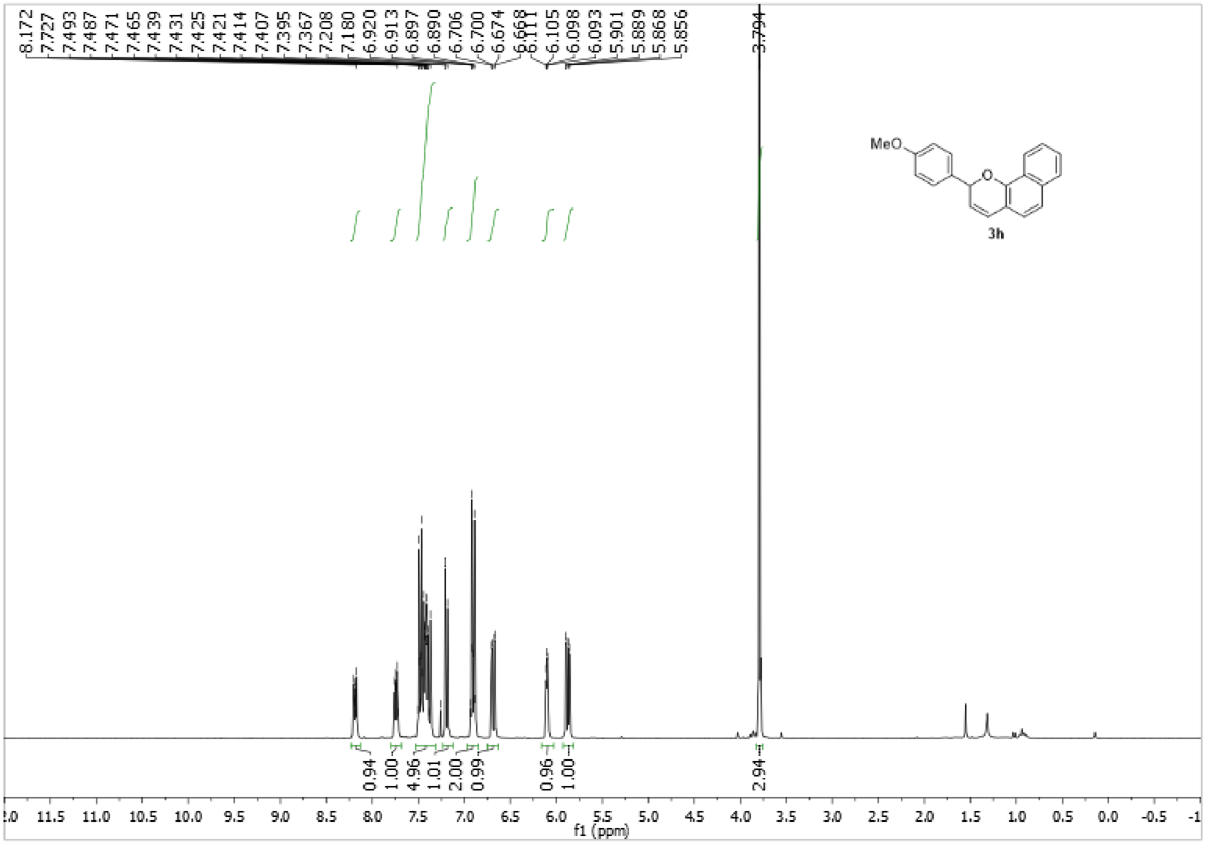
8.172
7.727
7.493
7.487
7.471
7.465
7.439
7.431
7.425
7.421
7.414
7.407
7.395
7.367
7.208
7.180
6.920
6.913
6.897
6.890
6.706
6.700
6.674
6.668
6.111
6.105
6.098
6.093
5.901
5.889
5.868
5.856
3.794
MeO
O
3h
0.94
1.00
4.96
1.01
2.00
0.99
0.96
1.00
2.94
f1 (ppm)

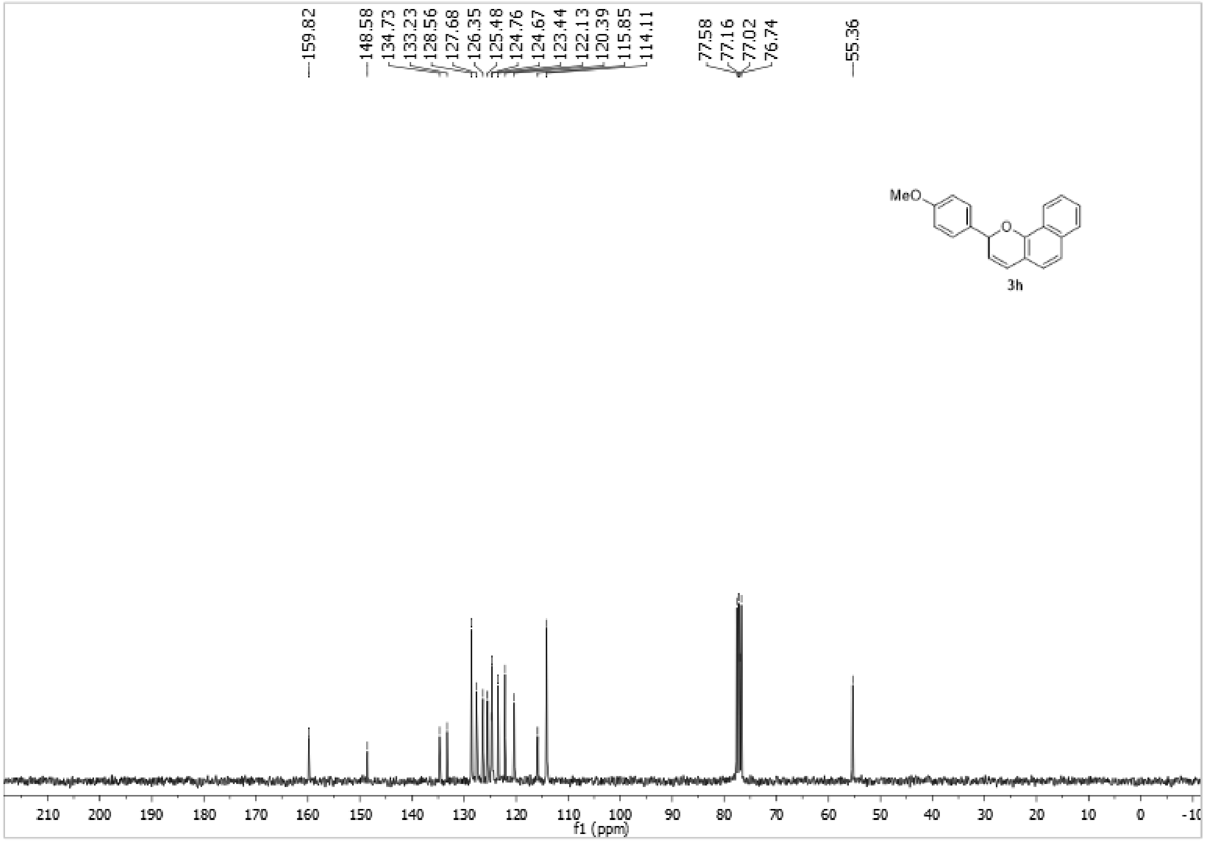
159.82
148.58
134.73
133.23
128.56
127.68
126.35
125.48
124.76
124.67
123.44
122.13
120.39
115.85
114.11
77.58
77.16
77.02
76.74
55.36
MeO
O
3h
f1 (ppm)

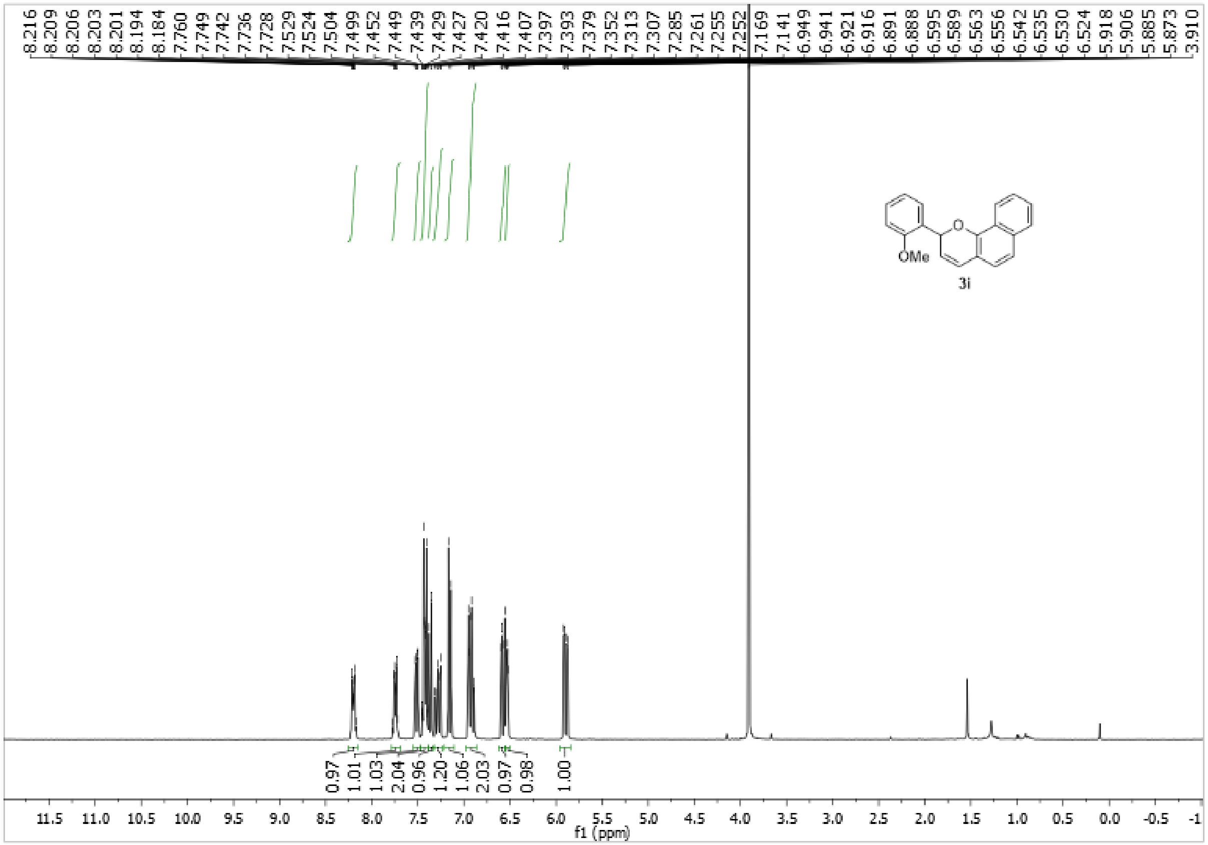
OMe
3i
f1 (ppm)

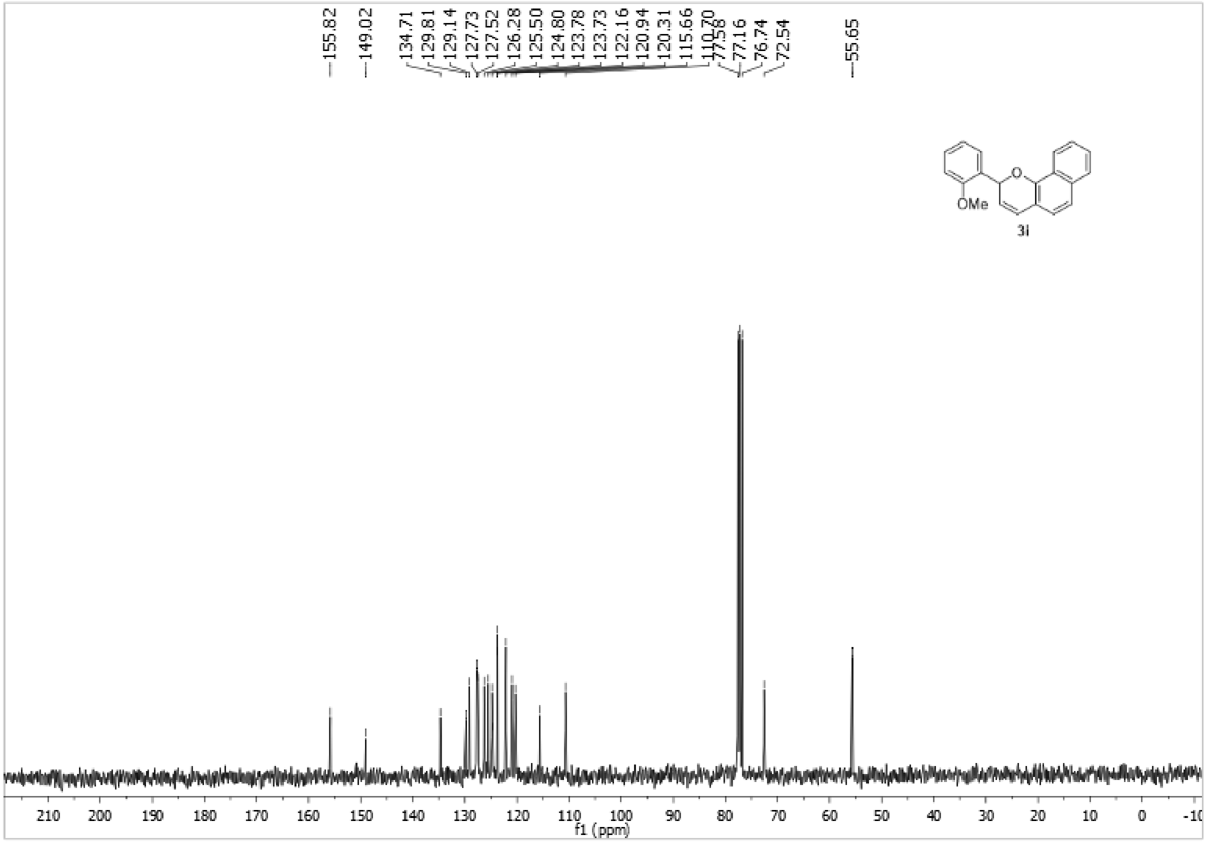
OMe
3i
f1 (ppm)

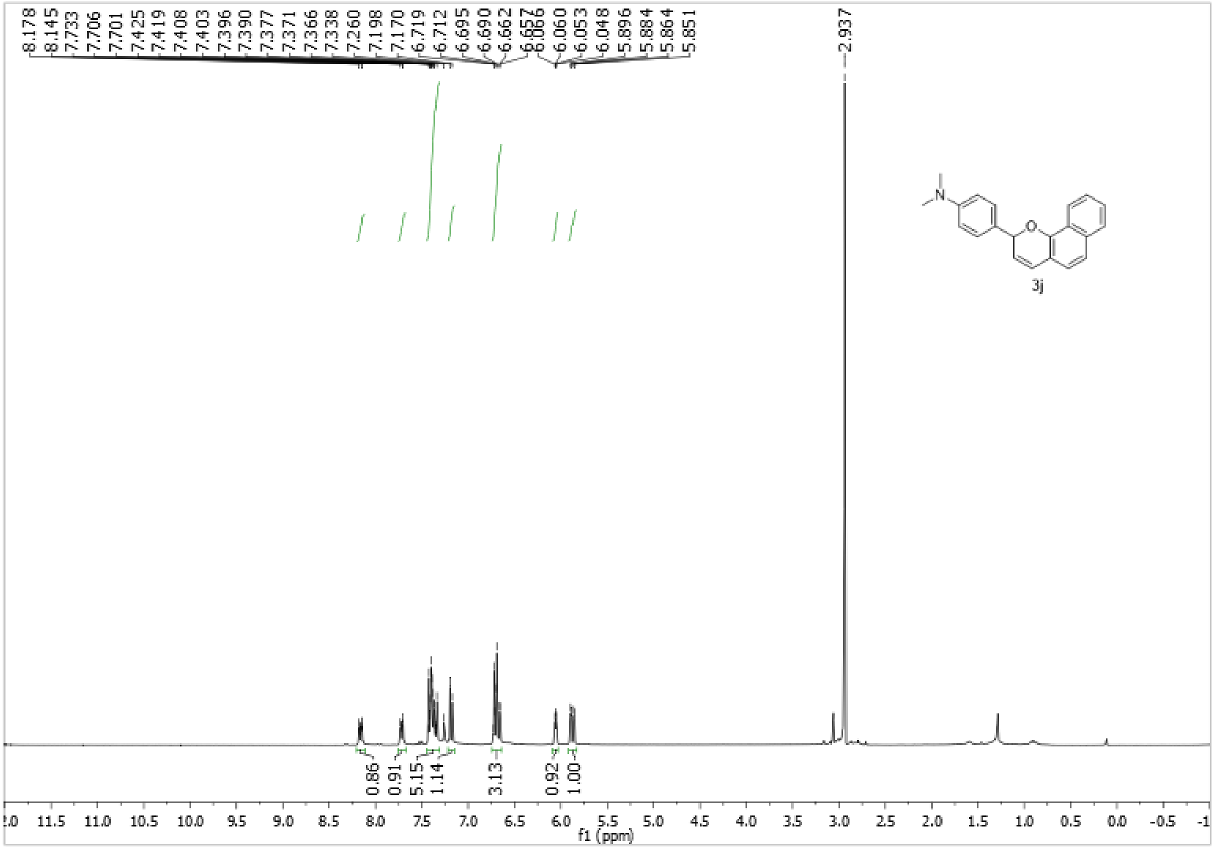

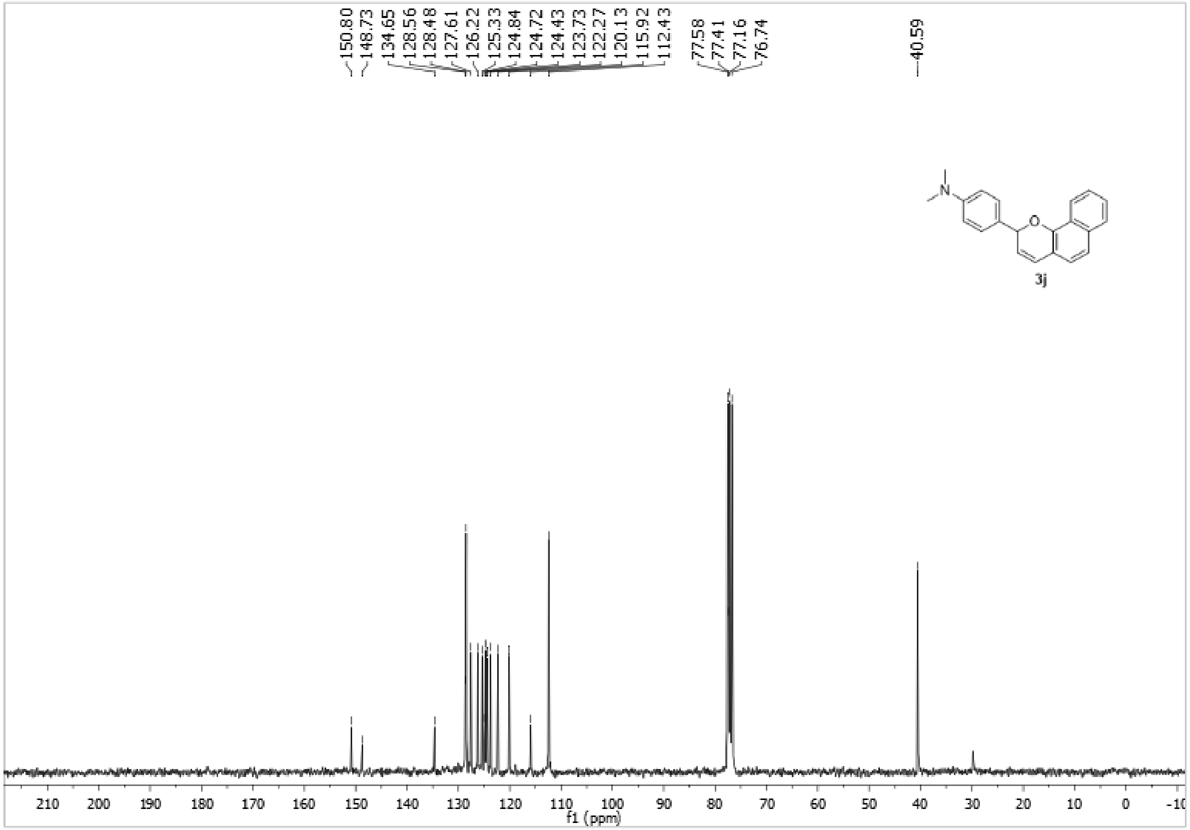

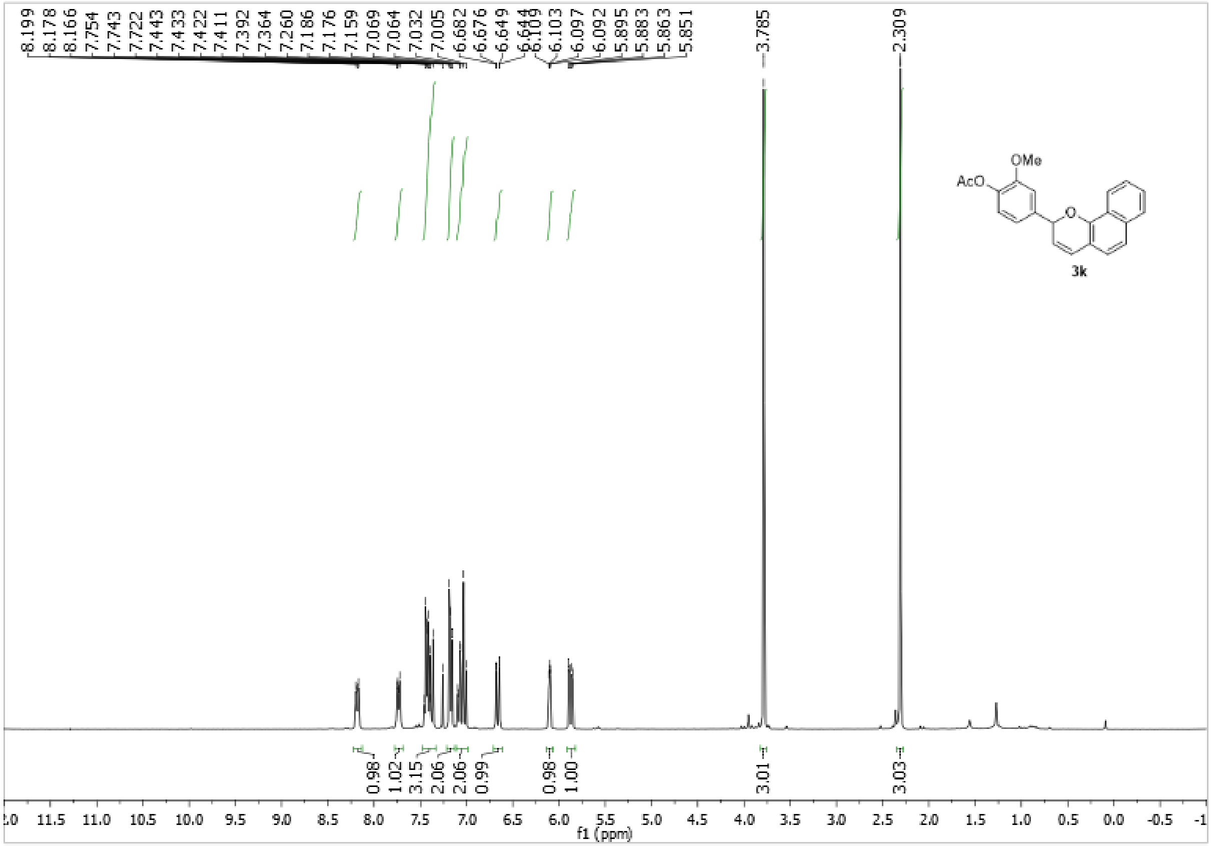

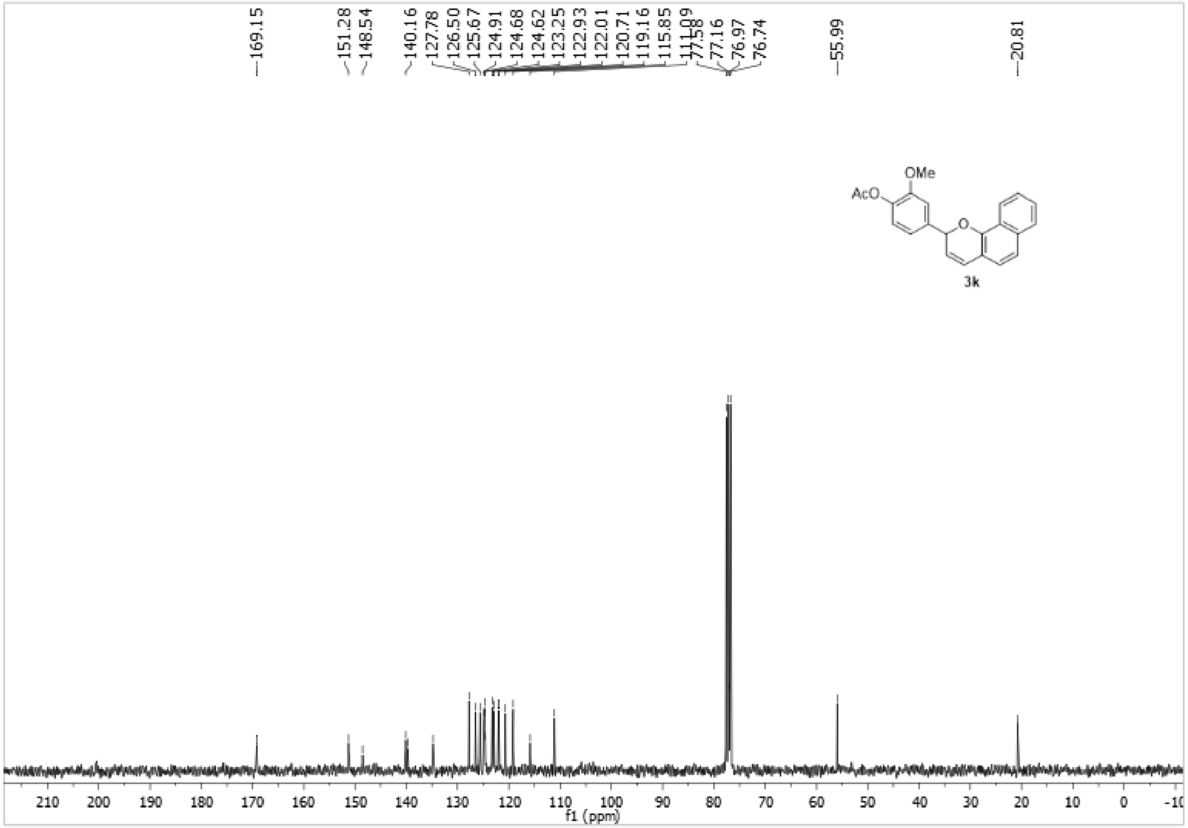

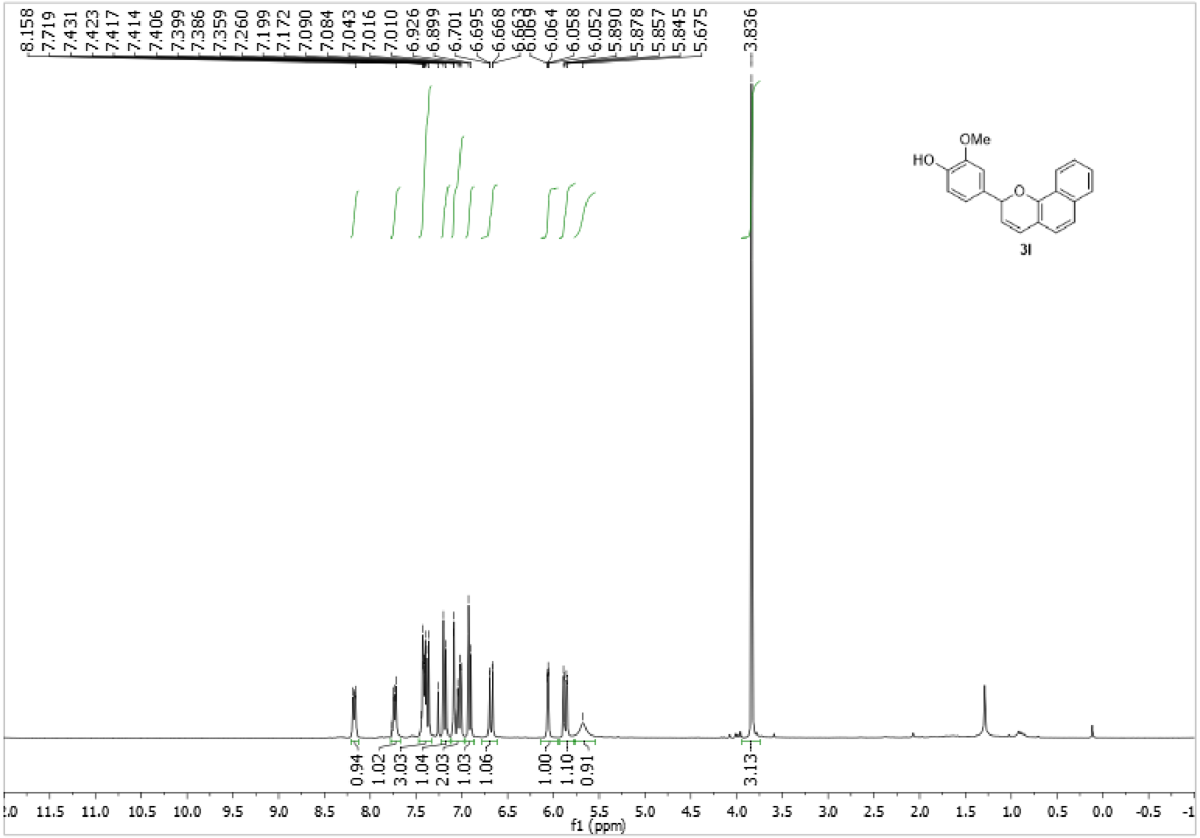
8.158
7.719
7.431
7.423
7.417
7.414
7.406
7.399
7.386
7.359
7.260
7.199
7.172
7.090
7.084
7.043
7.016
7.010
6.926
6.899
6.701
6.695
6.668
6.069
6.064
6.058
6.052
5.890
5.878
5.857
5.845
5.675
3.836
OMe
HO
O
3l
0.94
1.02
3.03
1.04
2.03
1.03
1.06
1.00
1.10
0.91
3.13
11.5 11.0 10.5 10.0 9.5 9.0 8.5 8.0 7.5 7.0 6.5 6.0 5.5 5.0 4.5 4.0 3.5 3.0 2.5 2.0 1.5 1.0 0.5 0.0 -0.5
f1 (ppm)

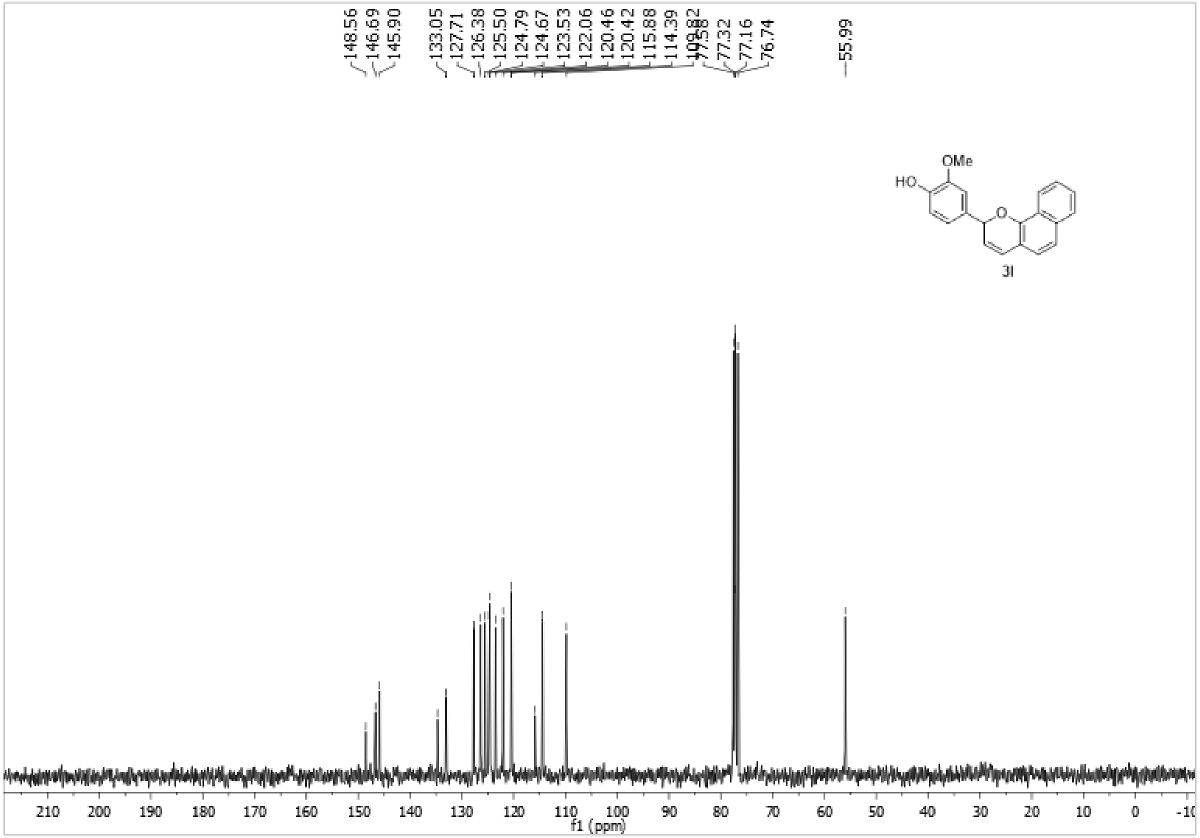
148.56
146.69
145.90
133.05
127.71
126.38
125.50
124.79
124.67
123.53
122.06
120.46
120.42
115.88
114.39
77.58
77.32
77.16
76.74
55.99
OMe
HO
O
3l
210 200 190 180 170 160 150 140 130 120 110 100 90 80 70 60 50 40 30 20 10 0
f1 (ppm)

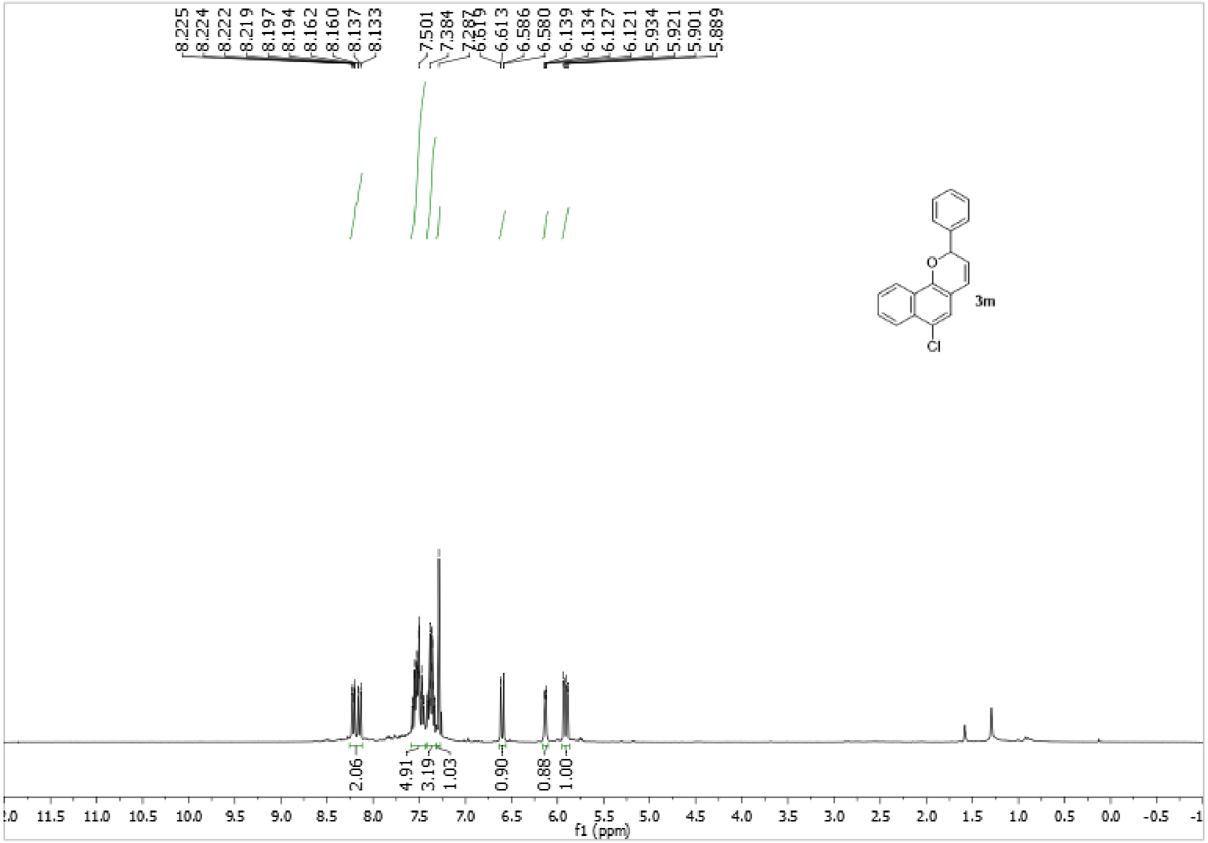

3m
Cl
O
f1 (ppm)

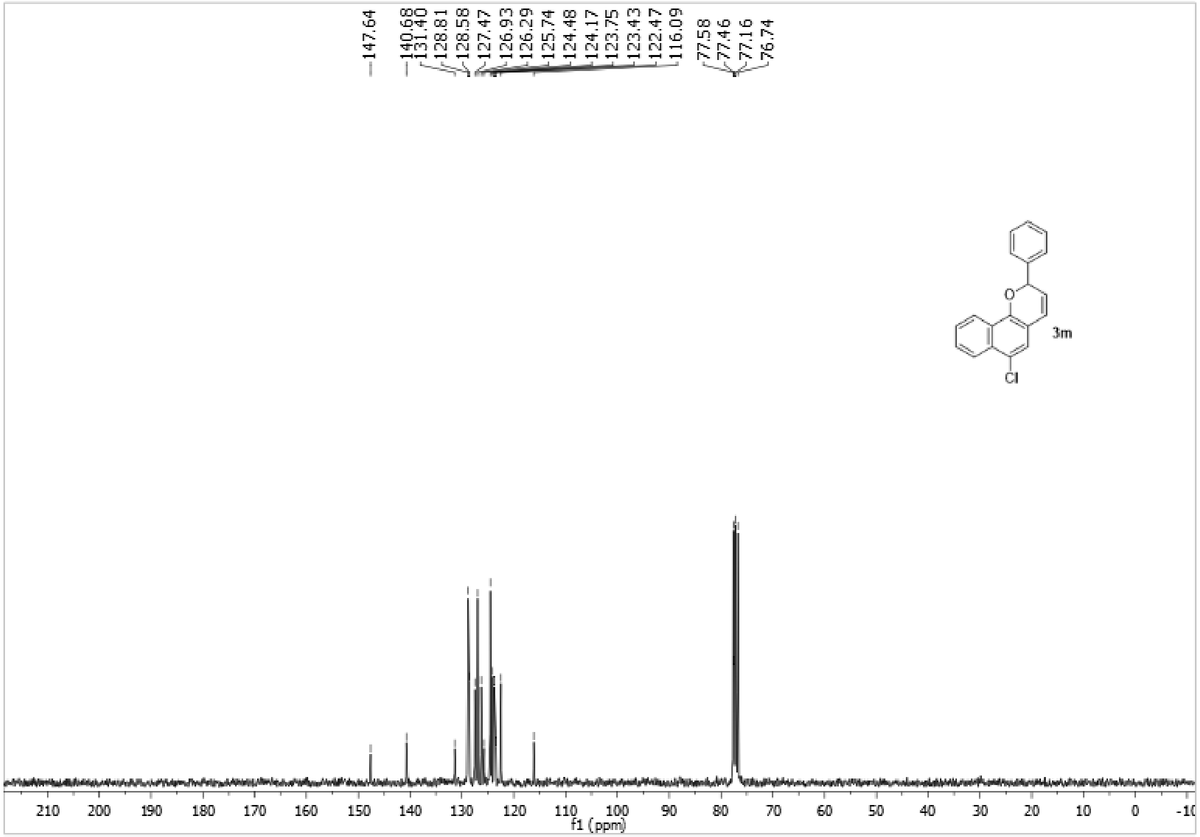

3m
Cl
O
f1 (ppm)

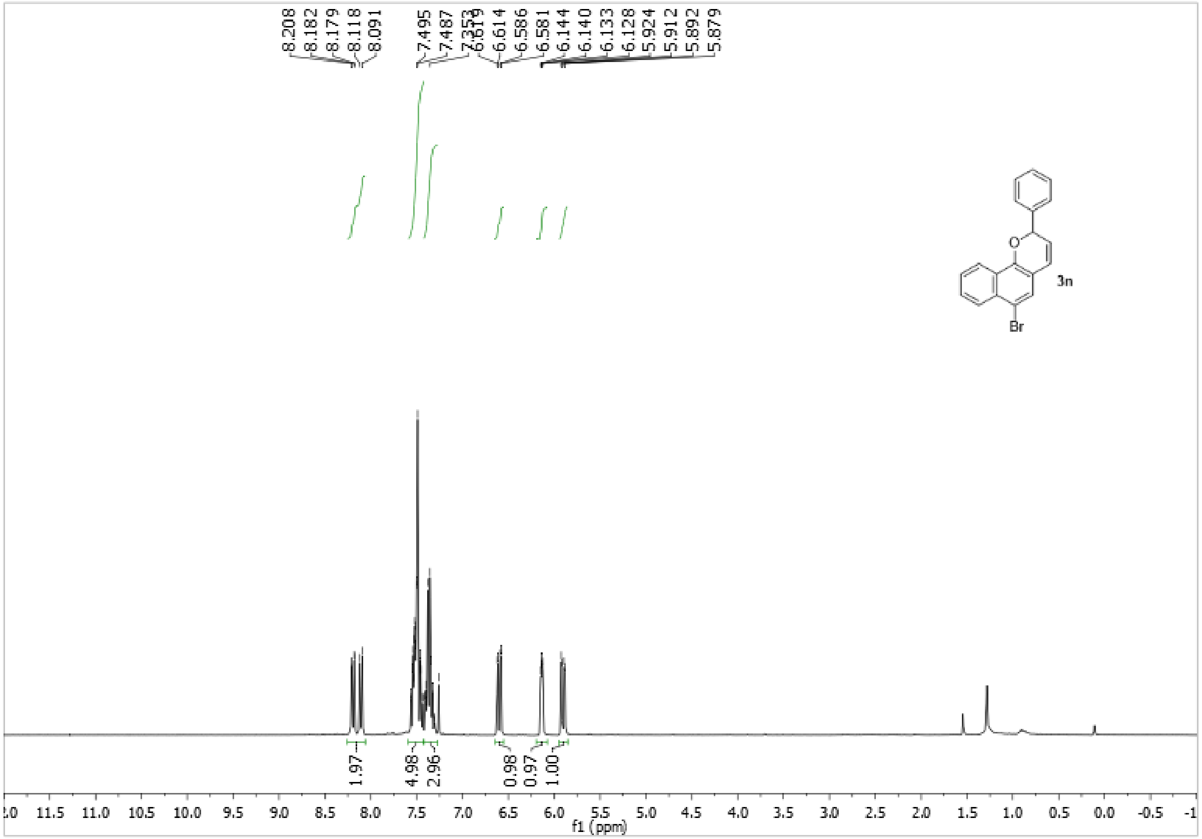
8.208
8.182
8.179
8.118
8.091
7.495
7.487
6.614
6.586
6.581
6.144
6.140
6.133
6.128
5.924
5.912
5.892
5.879
O
3n
Br
1.97
4.98
2.96
0.98
0.97
1.00
f1 (ppm)

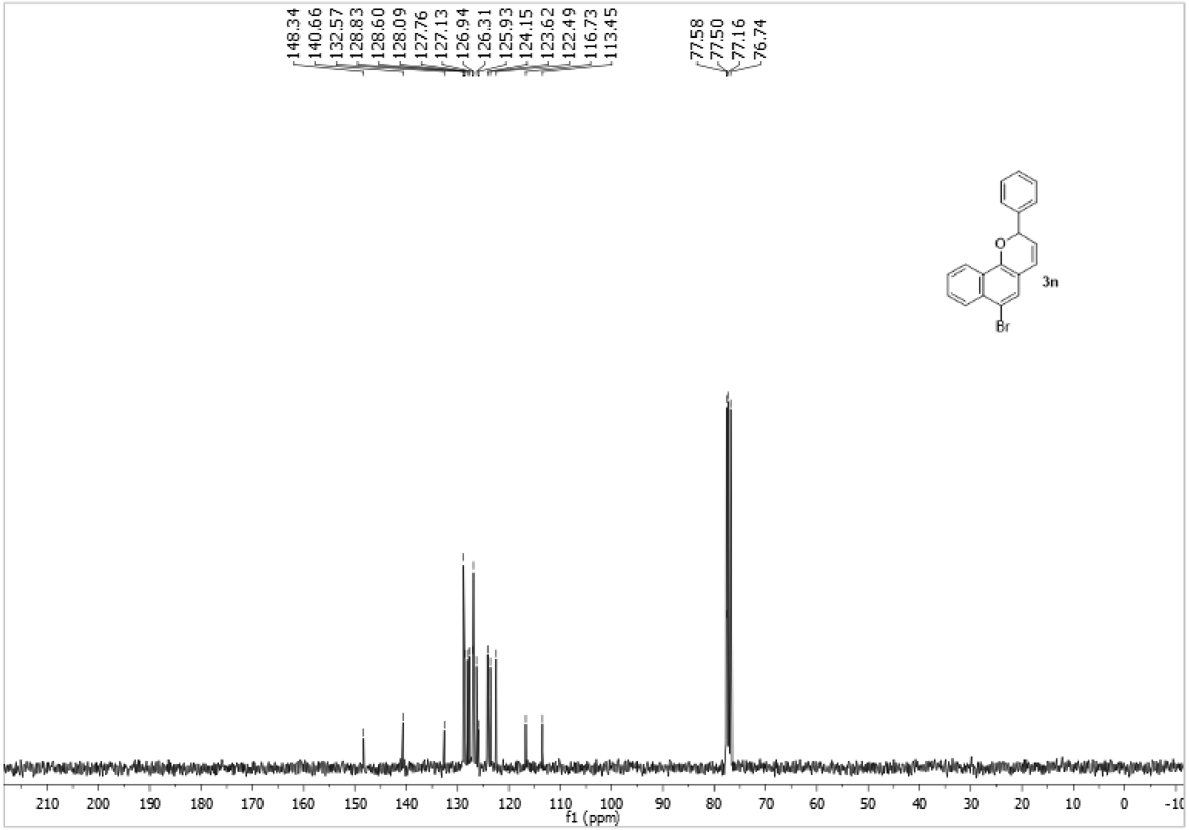
148.34
140.66
132.57
128.83
128.60
128.09
127.76
127.13
126.94
126.31
125.93
124.15
123.62
122.49
116.73
113.45
77.58
77.50
77.16
76.74
O
3n
Br
f1 (ppm)

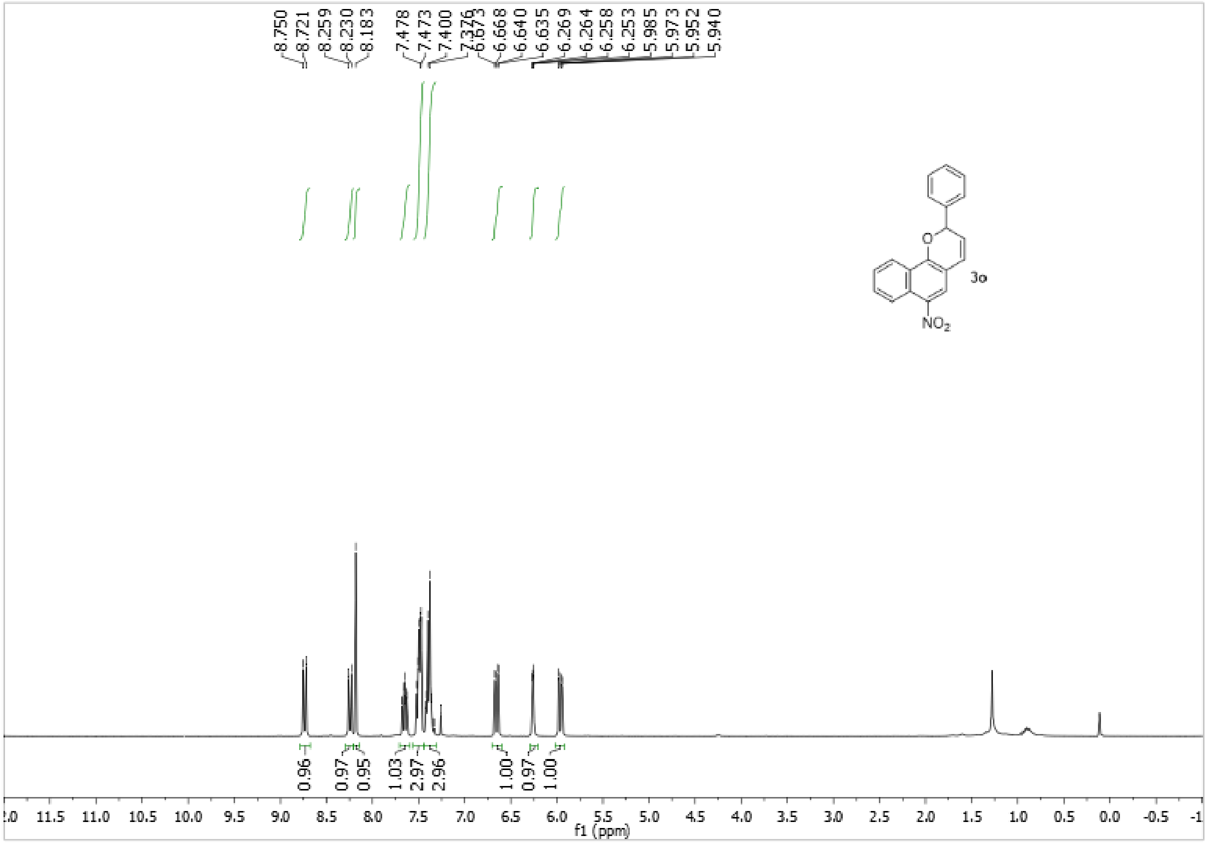
8.750
8.721
8.259
8.230
8.183
7.478
7.473
7.400
7.376
6.673
6.668
6.640
6.635
6.269
6.264
6.258
6.253
5.985
5.973
5.952
5.940
3o
0.96
0.97
0.95
1.03
2.97
2.96
1.00
0.97
1.00
f1 (ppm)

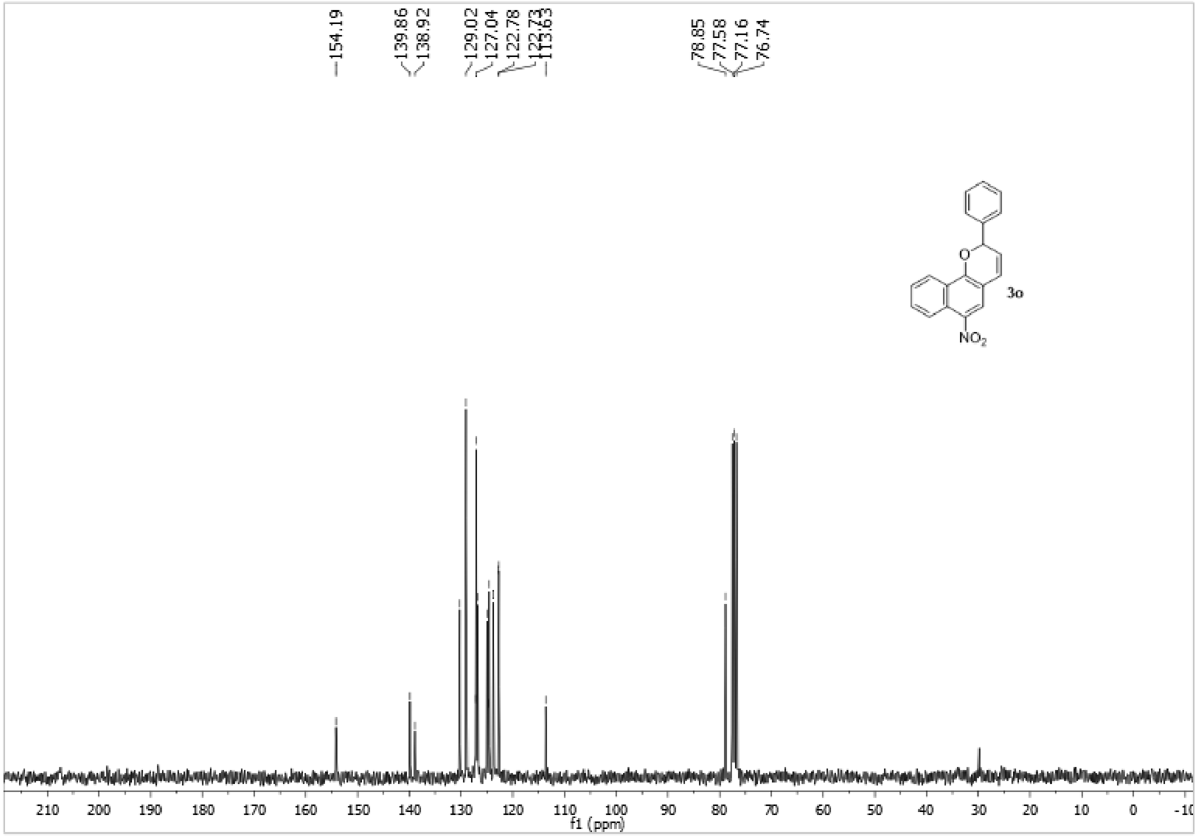
154.19
139.86
138.92
129.02
127.04
122.78
122.73
113.63
78.85
77.58
77.16
76.74
3o
f1 (ppm)

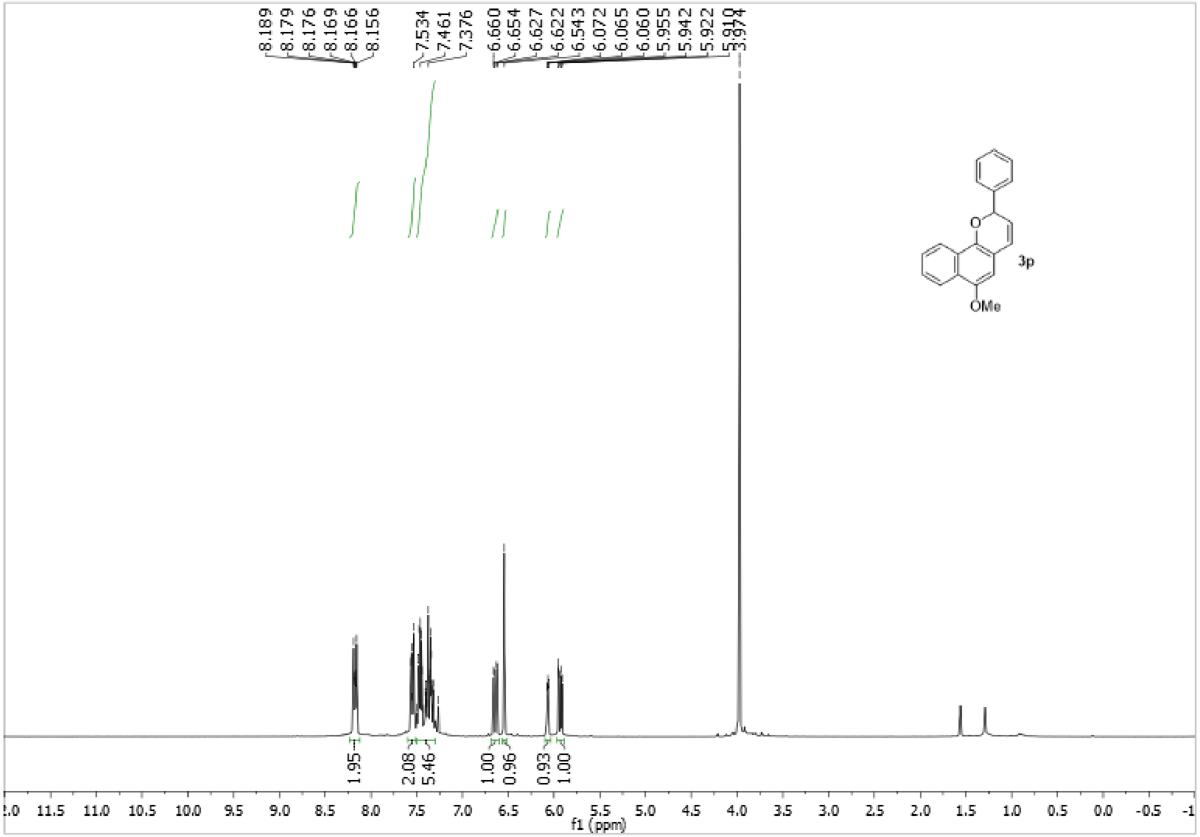
8.189
8.179
8.176
8.169
8.166
8.156
7.534
7.461
7.376
6.660
6.654
6.627
6.622
6.543
6.072
6.065
6.060
5.955
5.942
5.922
5.910
3.974
O
3p
OMe
1.95
2.08
5.46
1.00
0.96
0.93
1.00
f1 (ppm)

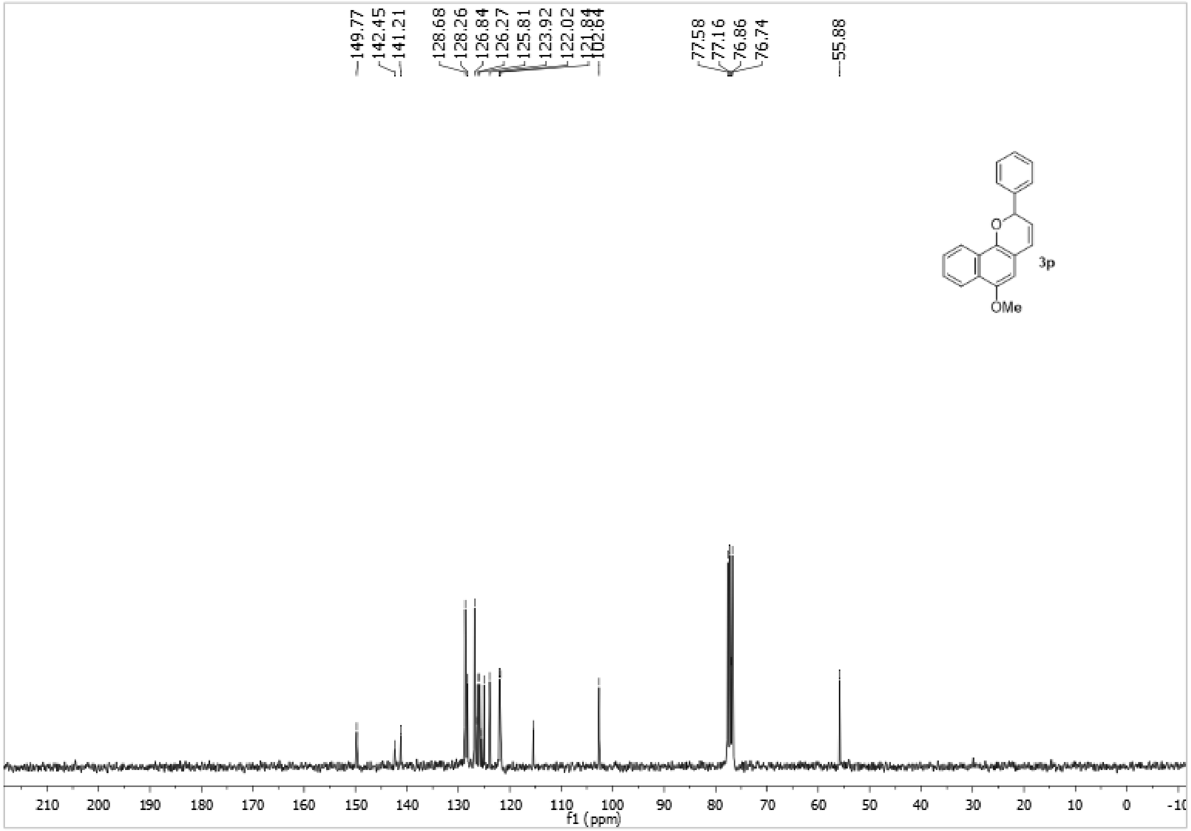
149.77
142.45
141.21
128.68
128.26
126.84
126.27
125.81
123.92
122.02
121.84
102.64
77.58
77.16
76.86
76.74
55.88
O
3p
OMe
f1 (ppm)

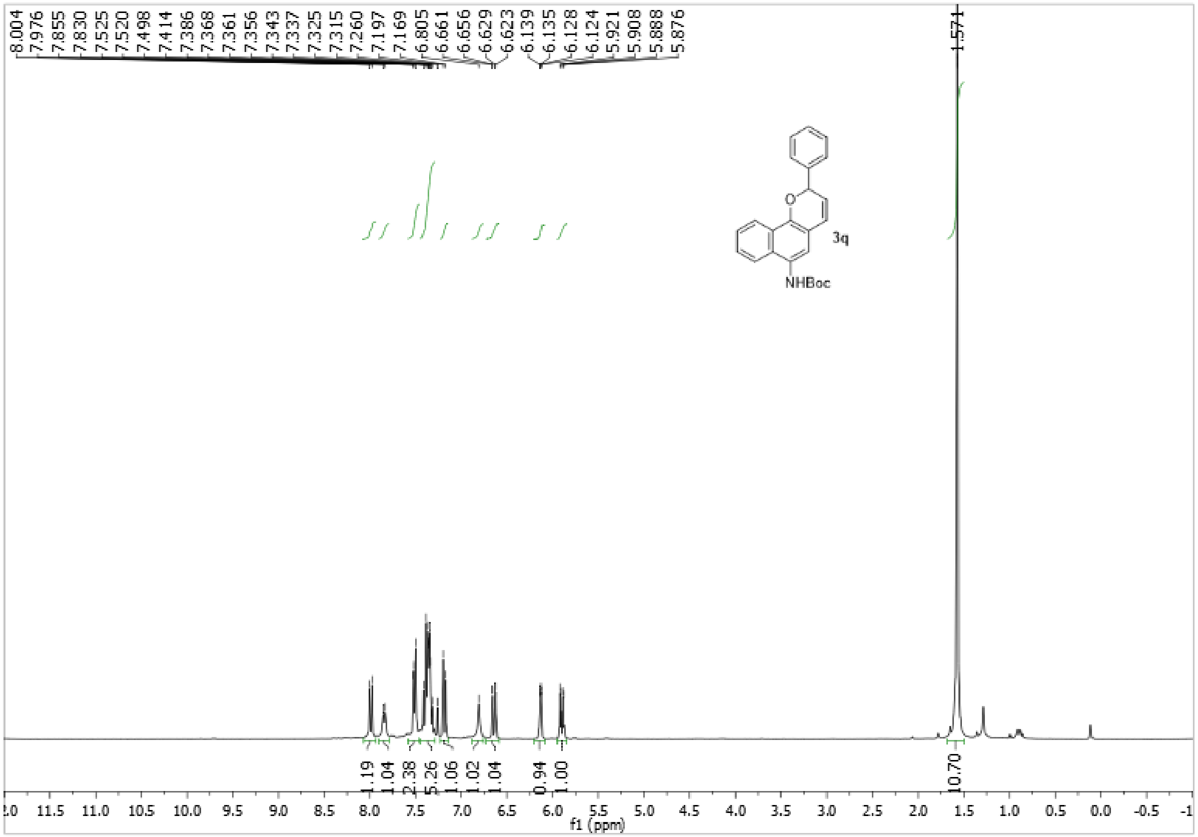
8.004 7.976 7.855 7.830 7.525 7.520 7.498 7.414 7.386 7.368 7.361 7.356 7.343 7.337 7.325 7.315 7.260 7.197 7.169 6.805 6.661 6.656 6.629 6.623 6.139 6.135 6.128 6.124 5.921 5.908 5.888 5.876
1.571
3q
NHBoc
1.19 1.04 2.38 5.26 1.06 1.02 1.04 0.94 1.00
10.70
f1 (ppm)

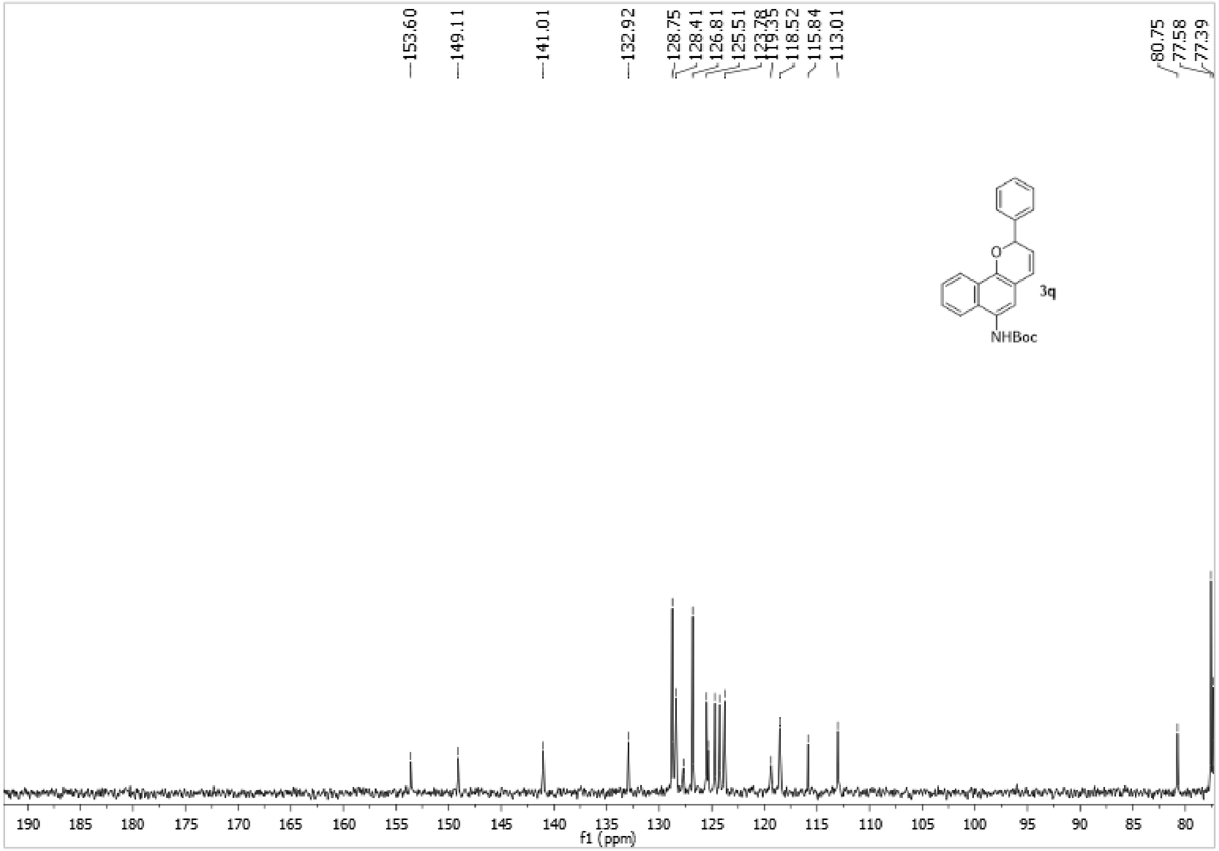
153.60 149.11 141.01 132.92 128.75 128.41 126.81 125.51 123.78 119.35 118.52 115.84 113.01
80.75 77.58 77.39
3q
NHBoc
f1 (ppm)

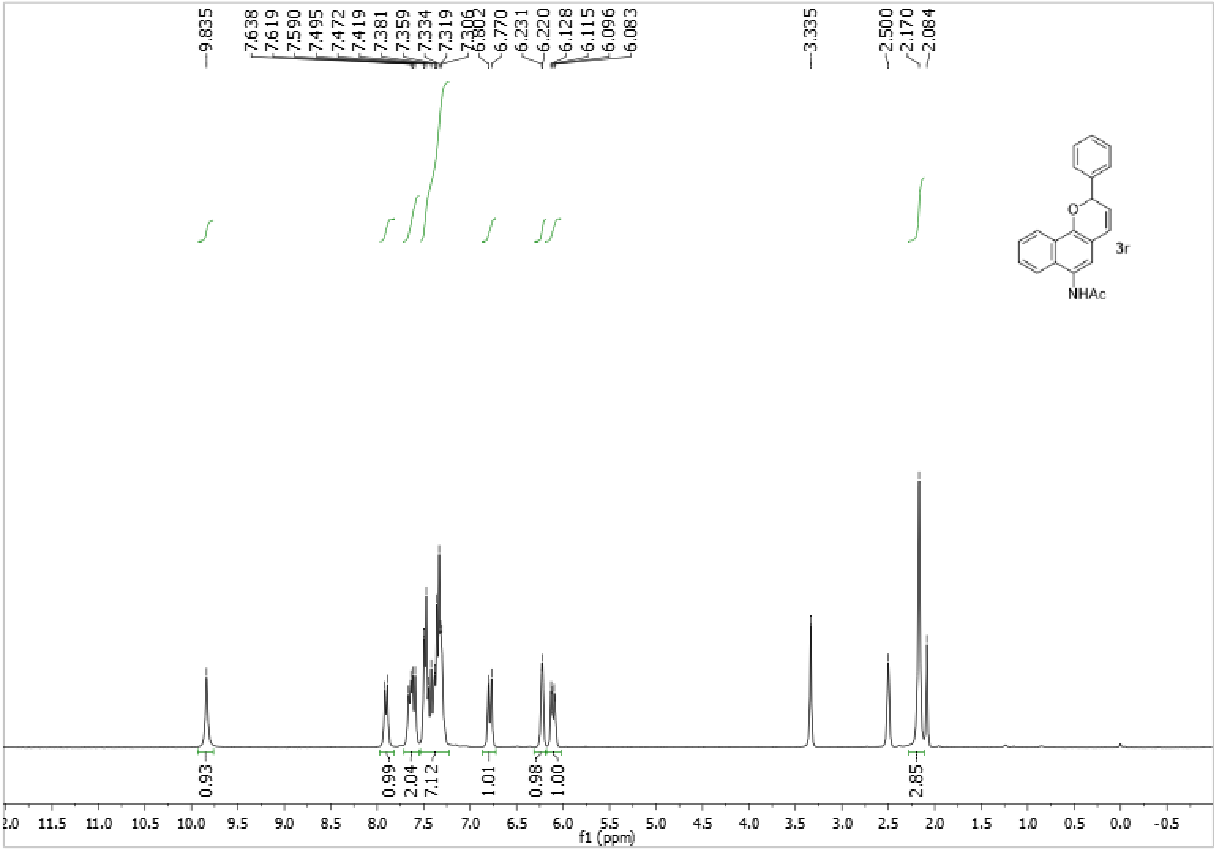
9.835
7.638
7.619
7.590
7.495
7.472
7.419
7.381
7.359
7.334
7.319
7.306
6.802
6.770
6.231
6.220
6.128
6.115
6.096
6.083
3.335
2.500
2.170
2.084
3r
NHAc
0.93
0.99
2.04
7.12
1.01
0.98
1.00
2.85
f1 (ppm)

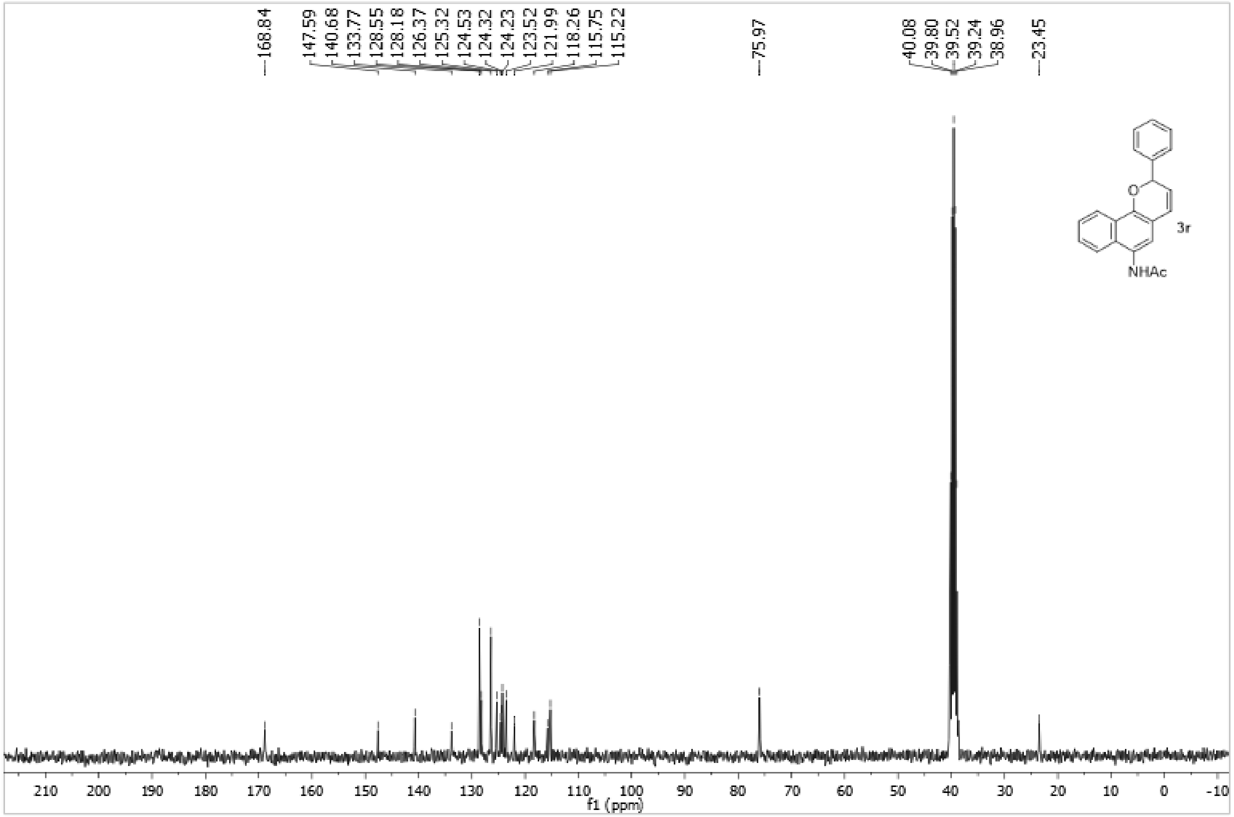
168.84
147.59
140.68
133.77
128.55
128.18
126.37
125.32
124.53
124.32
124.23
123.52
121.99
118.26
115.75
115.22
75.97
40.08
39.80
39.52
39.24
38.96
23.45
3r
NHAc
f1 (ppm)

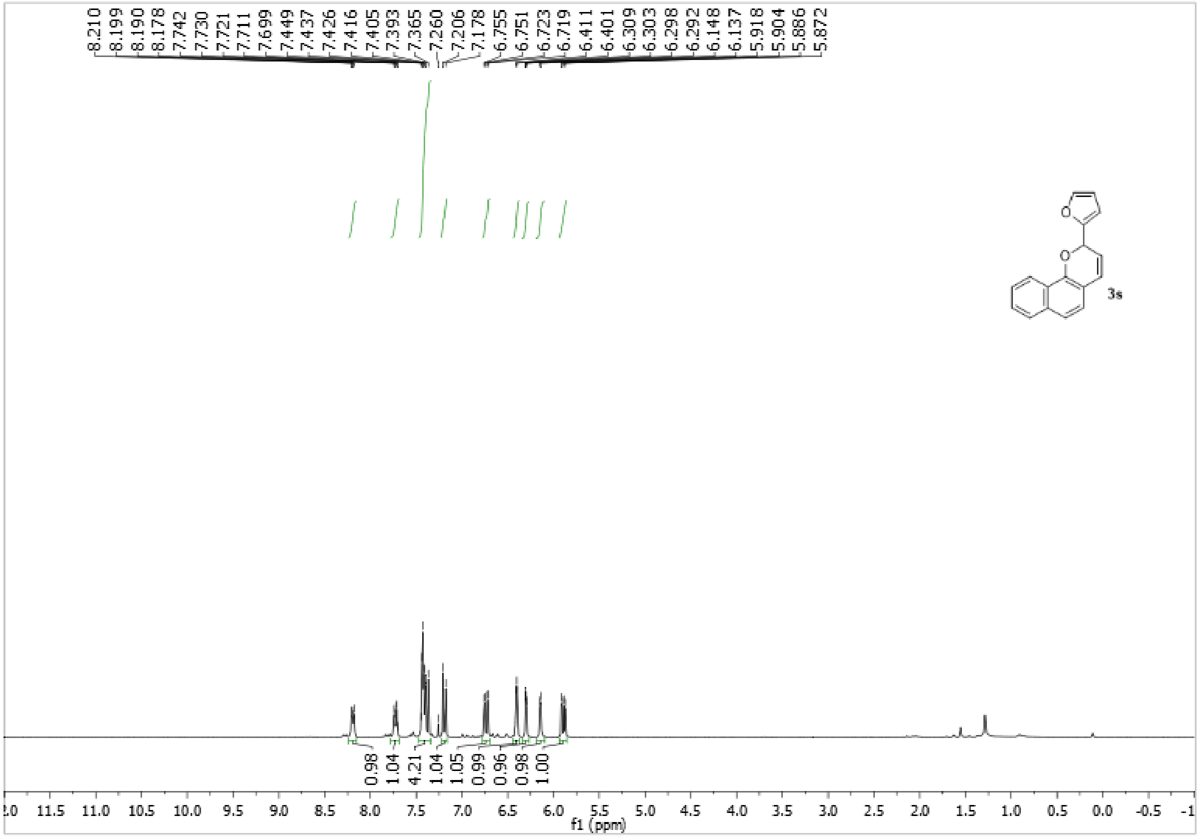

8.210
8.199
8.190
8.178
7.742
7.730
7.721
7.711
7.699
7.449
7.437
7.426
7.416
7.405
7.393
7.365
7.260
7.206
7.178
6.755
6.751
6.723
6.719
6.411
6.401
6.309
6.303
6.298
6.292
6.148
6.137
5.918
5.904
5.886
5.872
0.98
1.04
4.21
1.04
1.05
0.99
0.96
0.98
1.00
3s
f1 (ppm)

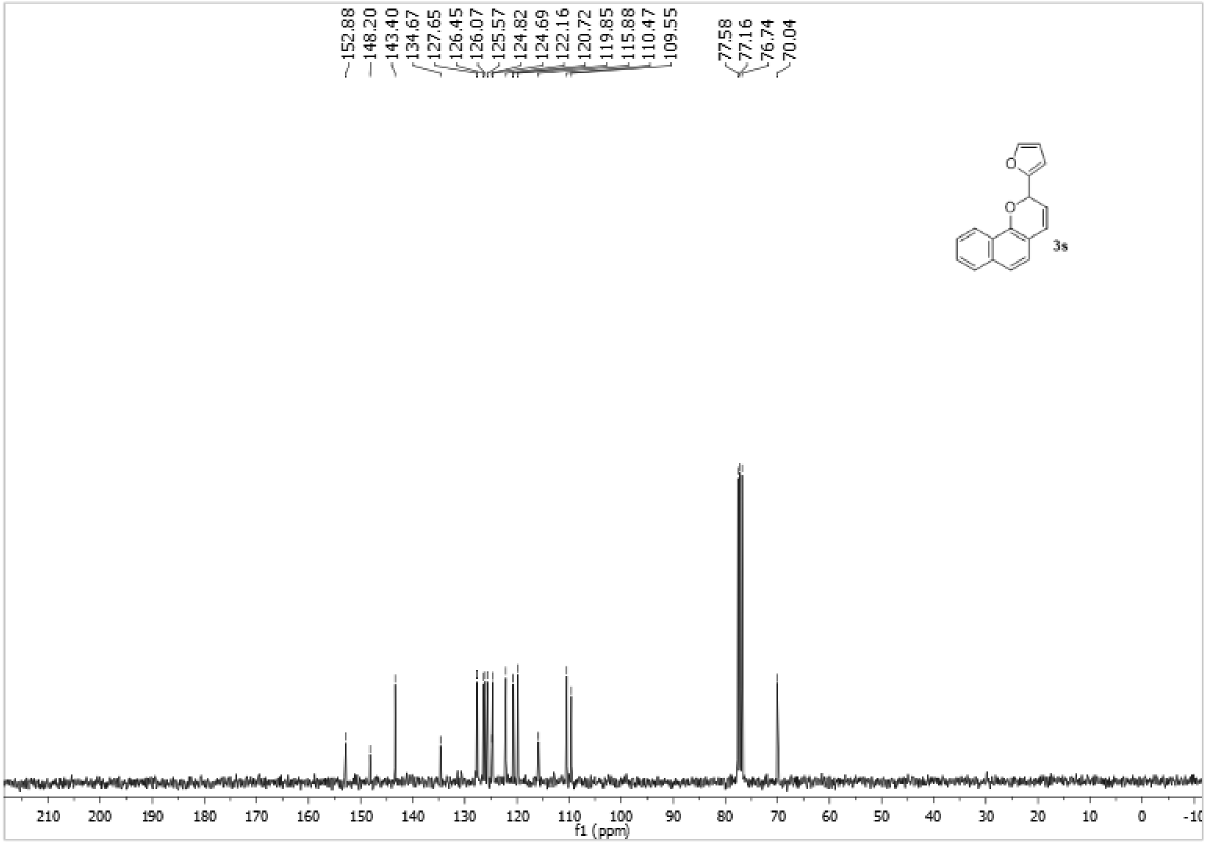

152.88
148.20
143.40
134.67
127.65
126.45
126.07
125.57
124.82
124.69
122.16
120.72
119.85
115.88
110.47
109.55
77.58
77.16
76.74
70.04
3s
f1 (ppm)

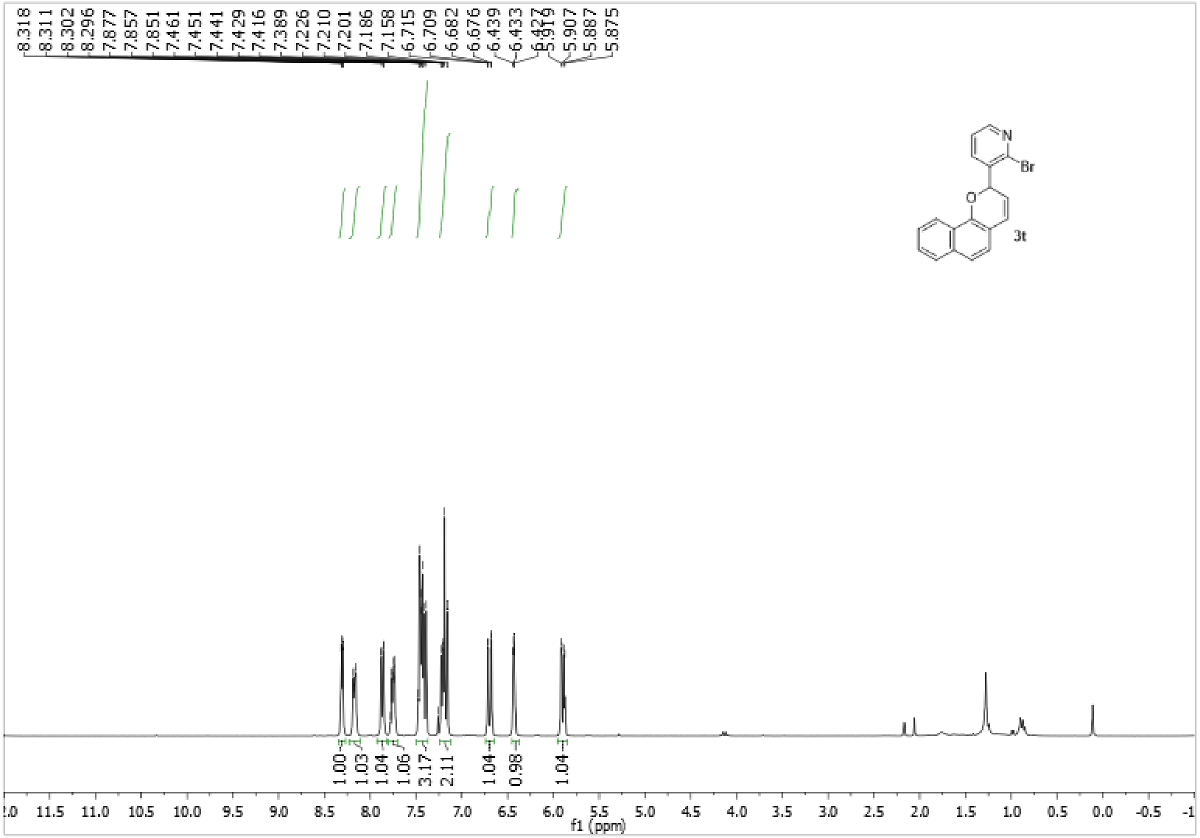
8.318
8.311
8.302
8.296
7.877
7.857
7.851
7.461
7.451
7.441
7.429
7.416
7.389
7.226
7.210
7.201
7.186
7.158
6.715
6.709
6.682
6.676
6.439
6.433
6.427
5.919
5.907
5.887
5.875
N
Br
O
3t
1.00
1.03
1.04
1.06
3.17
2.11
1.04
0.98
1.04
f1 (ppm)

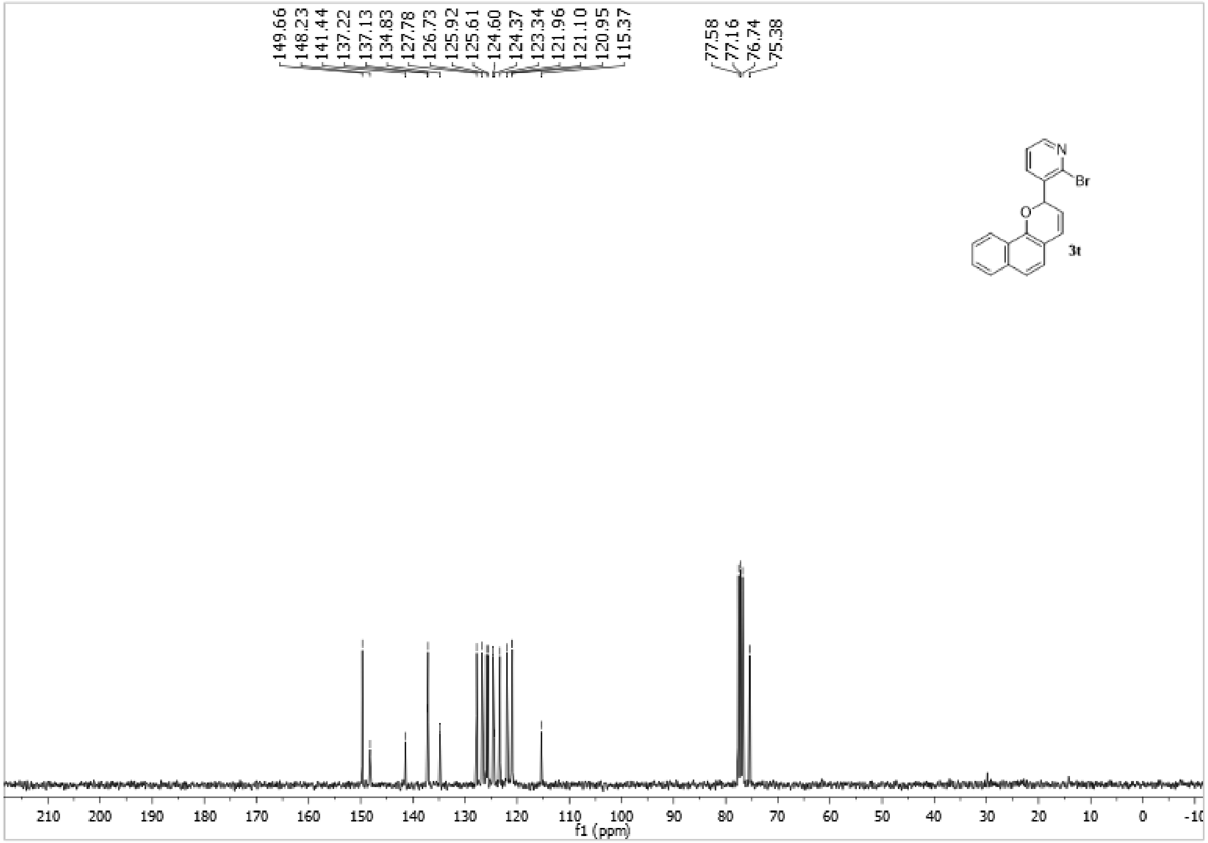
149.66
148.23
141.44
137.22
137.13
134.83
127.78
126.73
125.92
125.61
124.60
124.37
123.34
121.96
121.10
120.95
115.37
77.58
77.16
76.74
75.38
N
Br
O
3t
f1 (ppm)

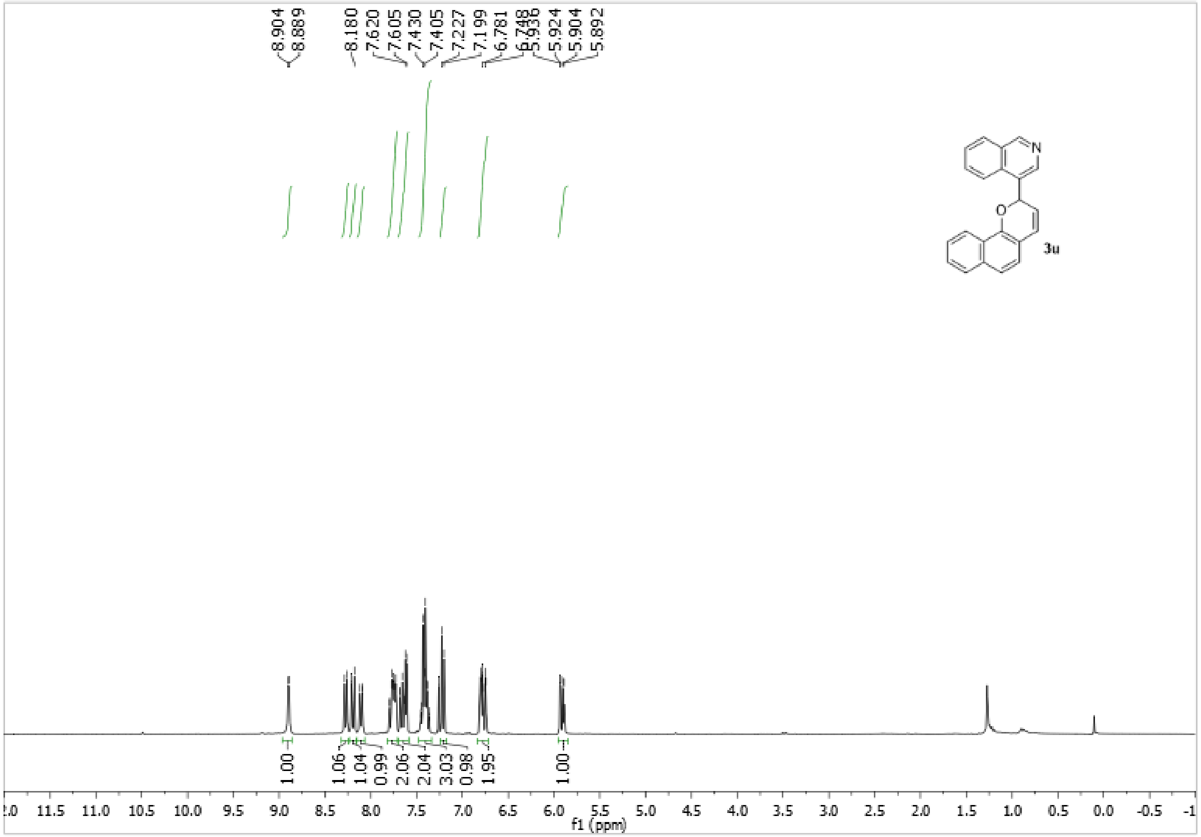
8.904
8.889
8.180
7.620
7.605
7.430
7.405
7.227
7.199
6.781
6.748
5.936
5.924
5.904
5.892
1.00
1.06
1.04
0.99
2.06
2.04
3.03
0.98
1.95
1.00
3u
f1 (ppm)

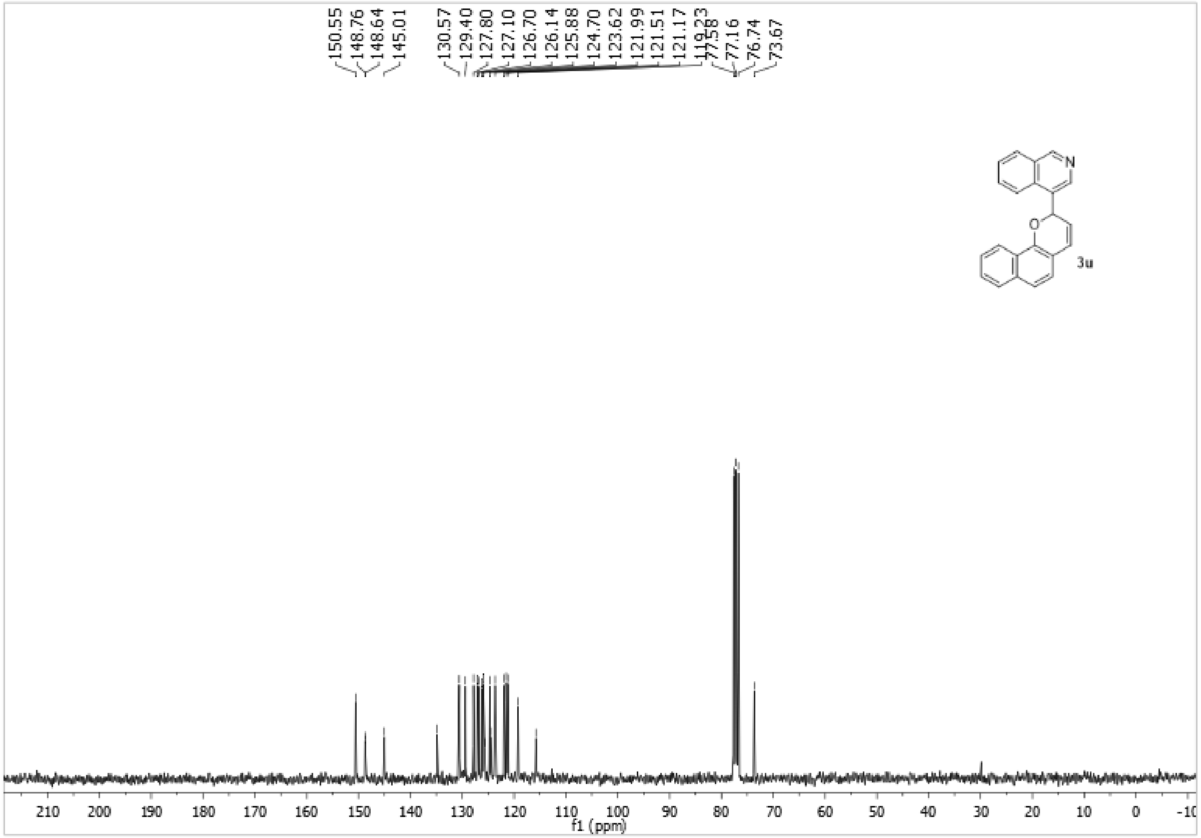
150.55
148.76
148.64
145.01
130.57
129.40
127.80
127.10
126.70
126.14
125.88
124.70
123.62
121.99
121.51
121.17
119.23
77.58
77.16
76.74
73.67
3u
f1 (ppm)

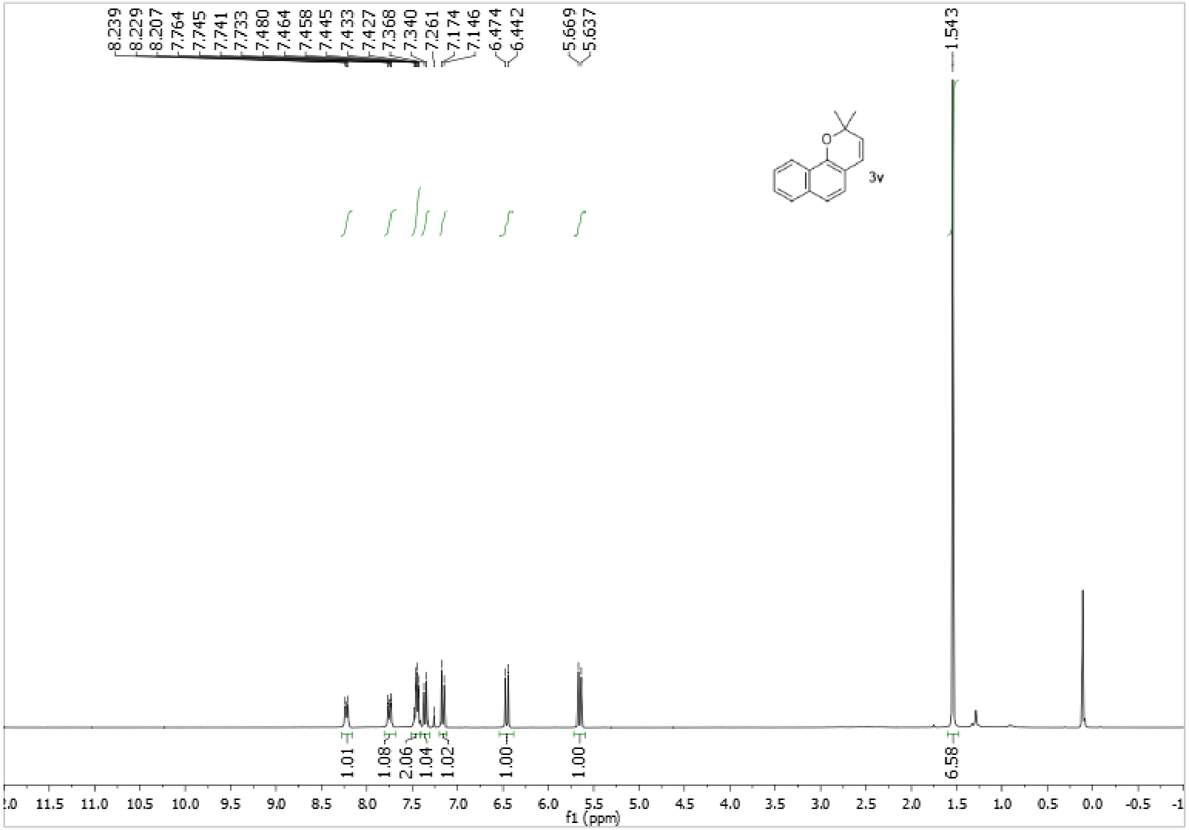
8.239
8.229
8.207
7.764
7.745
7.741
7.733
7.480
7.464
7.458
7.445
7.433
7.427
7.368
7.340
7.261
7.174
7.146
6.474
6.442
5.669
5.637
1.543
O
3v
1.01
1.08
2.06
1.04
1.02
1.00
1.00
6.58
f1 (ppm)

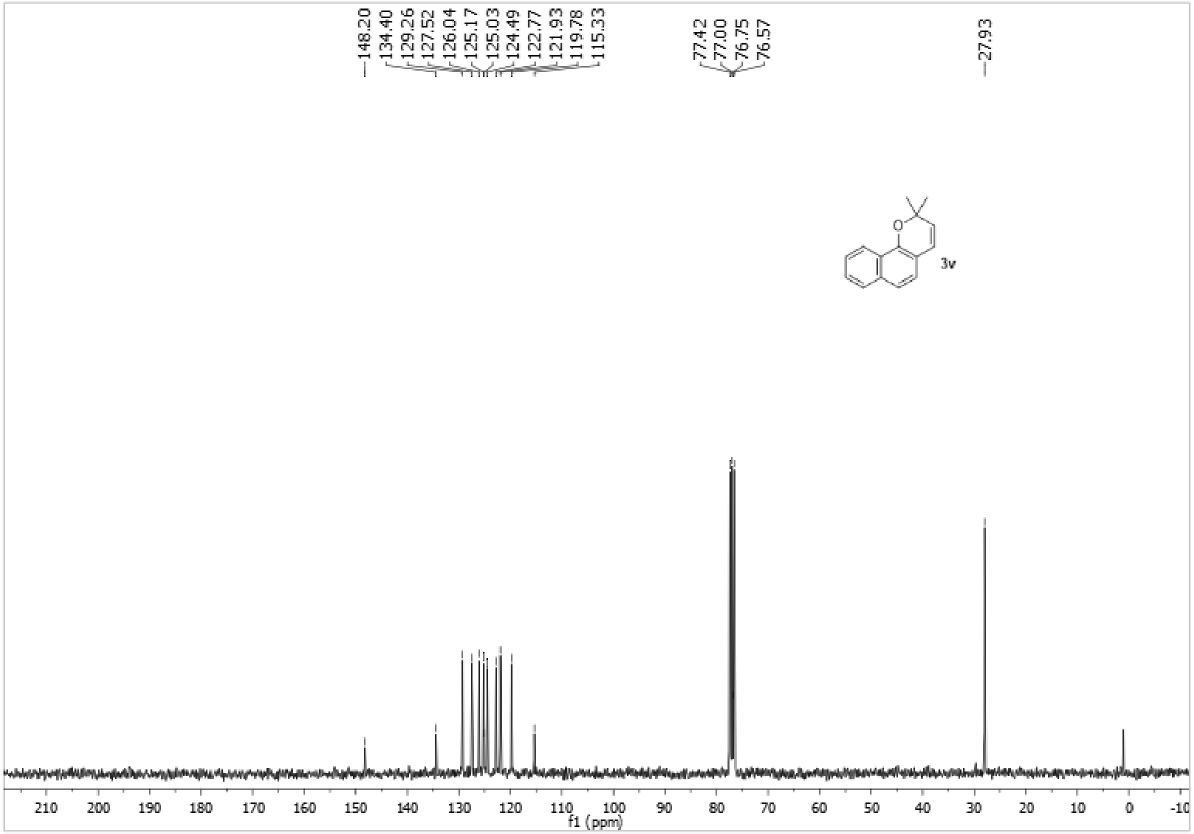
148.20
134.40
129.26
127.52
126.04
125.17
125.03
124.49
122.77
121.93
119.78
115.33
77.42
77.00
76.75
76.57
27.93
O
3v
f1 (ppm)

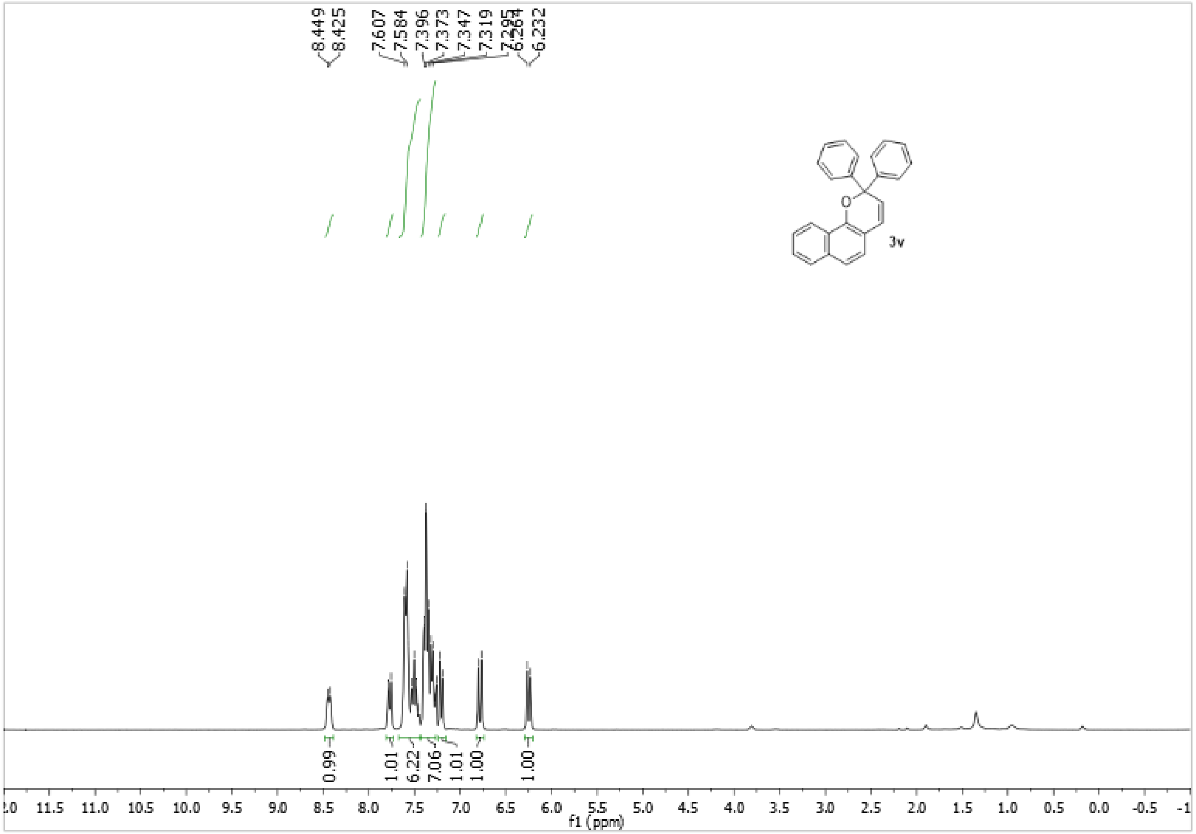
8.449
8.425
7.607
7.584
7.396
7.373
7.347
7.319
7.295
6.264
6.232
3v
0.99
1.01
6.22
7.06
1.01
1.00
1.00
f1 (ppm)

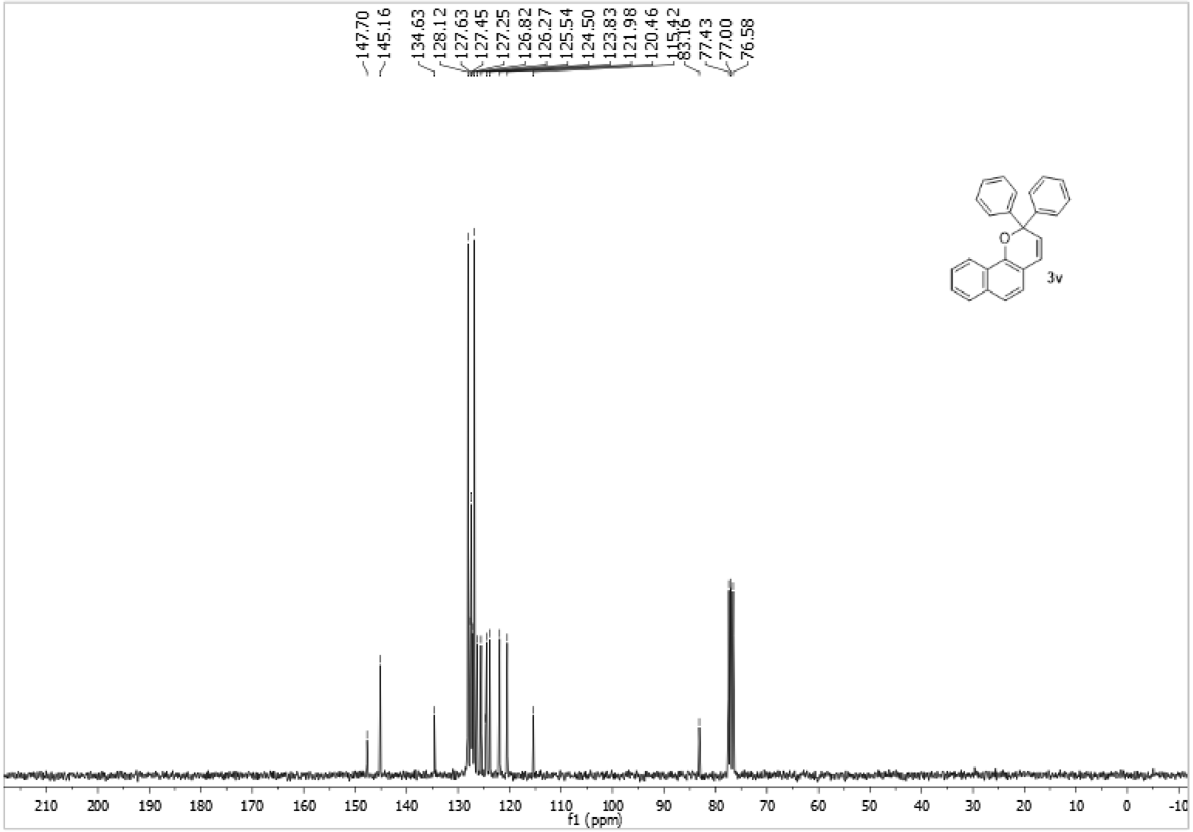
147.70
145.16
134.63
128.12
127.63
127.45
127.25
126.82
126.27
125.54
124.50
123.83
121.98
120.46
115.42
83.16
77.43
77.00
76.58
3v
f1 (ppm)

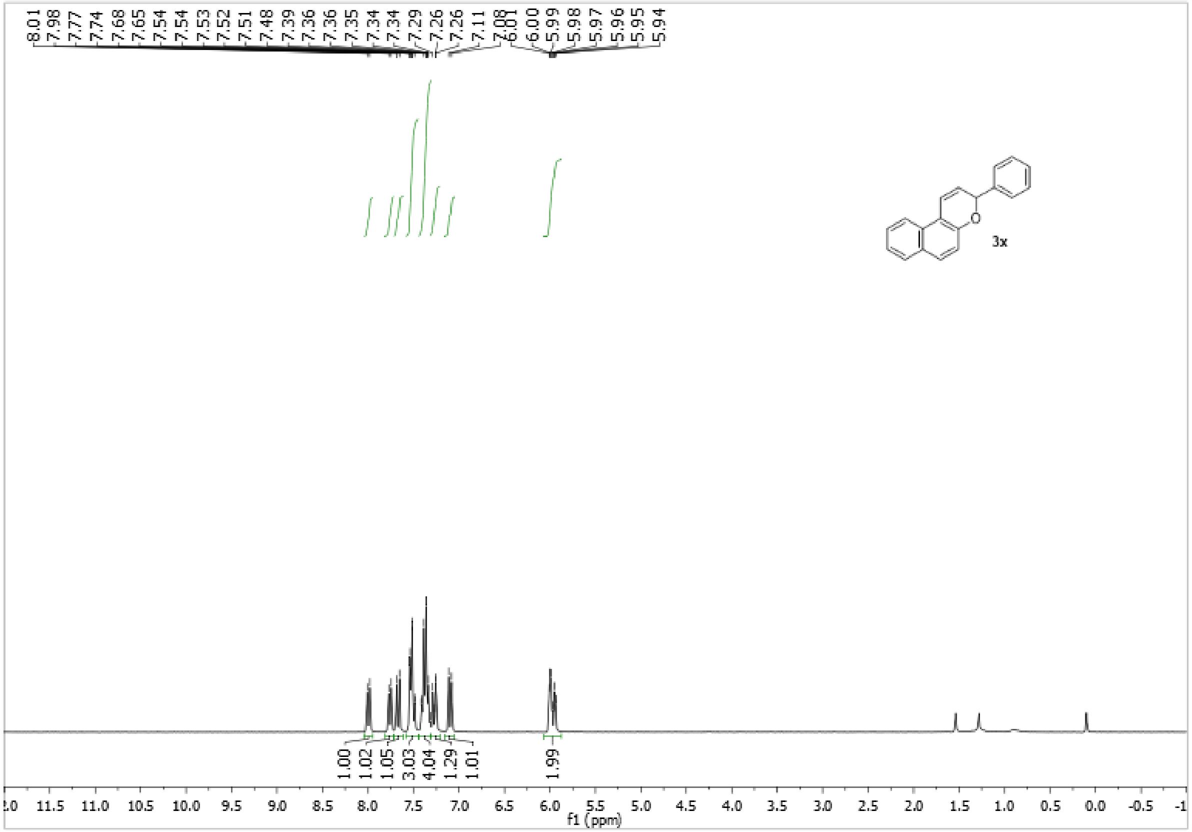

8.01 7.98 7.77 7.74 7.68 7.65 7.54 7.54 7.53 7.52 7.51 7.48 7.39 7.36 7.36 7.35 7.34 7.34 7.29 7.26 7.26 7.11 7.08 6.01 6.00 5.99 5.98 5.97 5.96 5.95 5.94
3x
1.00 1.02 1.05 3.03 4.04 1.29 1.01 1.99
f1 (ppm)

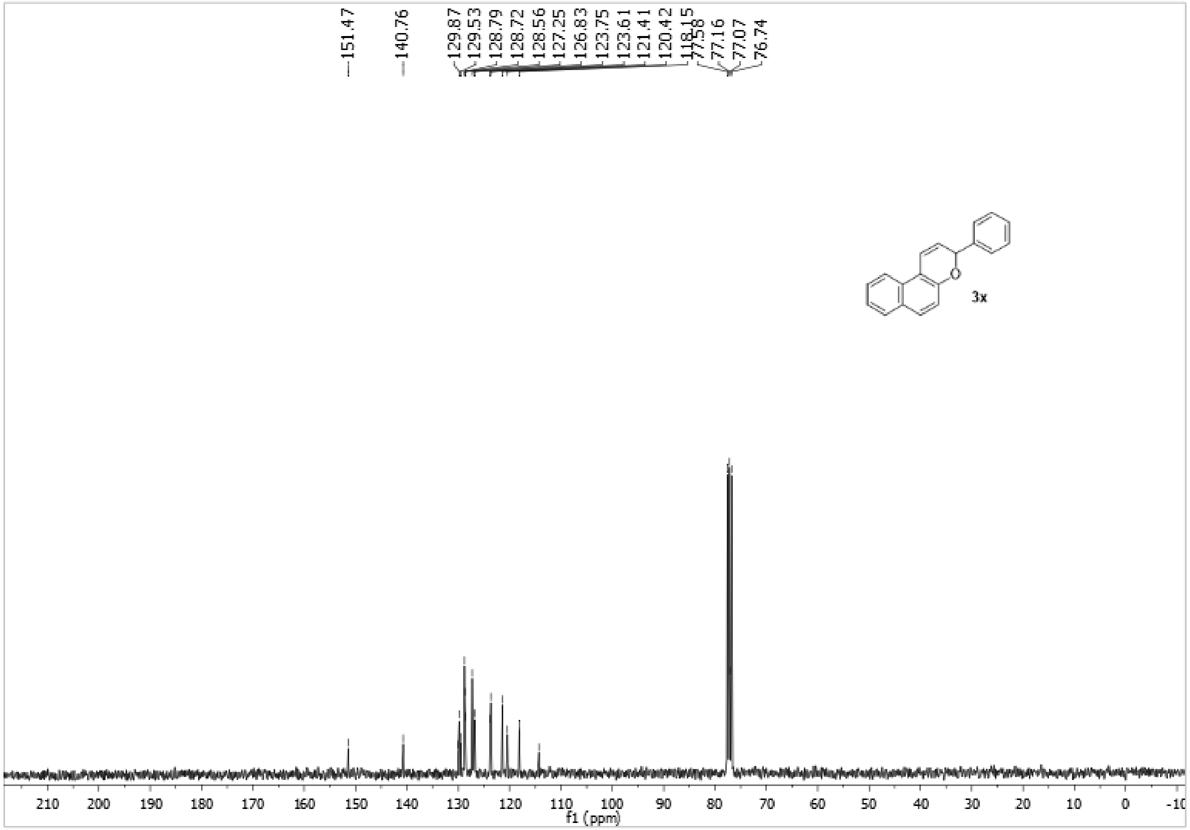

151.47 140.76 129.87 129.53 128.79 128.72 128.56 127.25 126.83 123.75 123.61 121.41 120.42 118.15 77.58 77.16 77.07 76.74
3x
f1 (ppm)

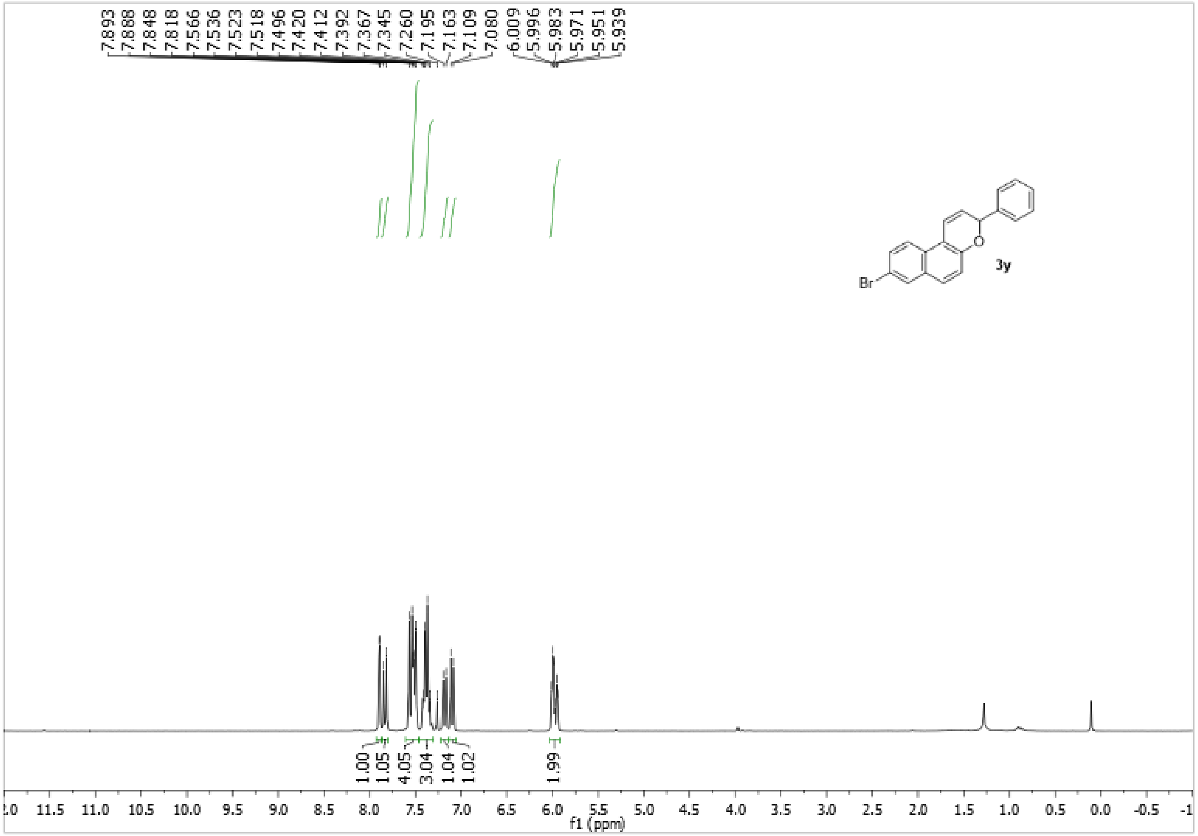
7.893
7.888
7.848
7.818
7.566
7.536
7.523
7.518
7.496
7.420
7.412
7.392
7.367
7.345
7.260
7.195
7.163
7.109
7.080
6.009
5.996
5.983
5.971
5.951
5.939
3y
Br
O
1.00
1.05
4.05
3.04
1.04
1.02
1.99
f1 (ppm)

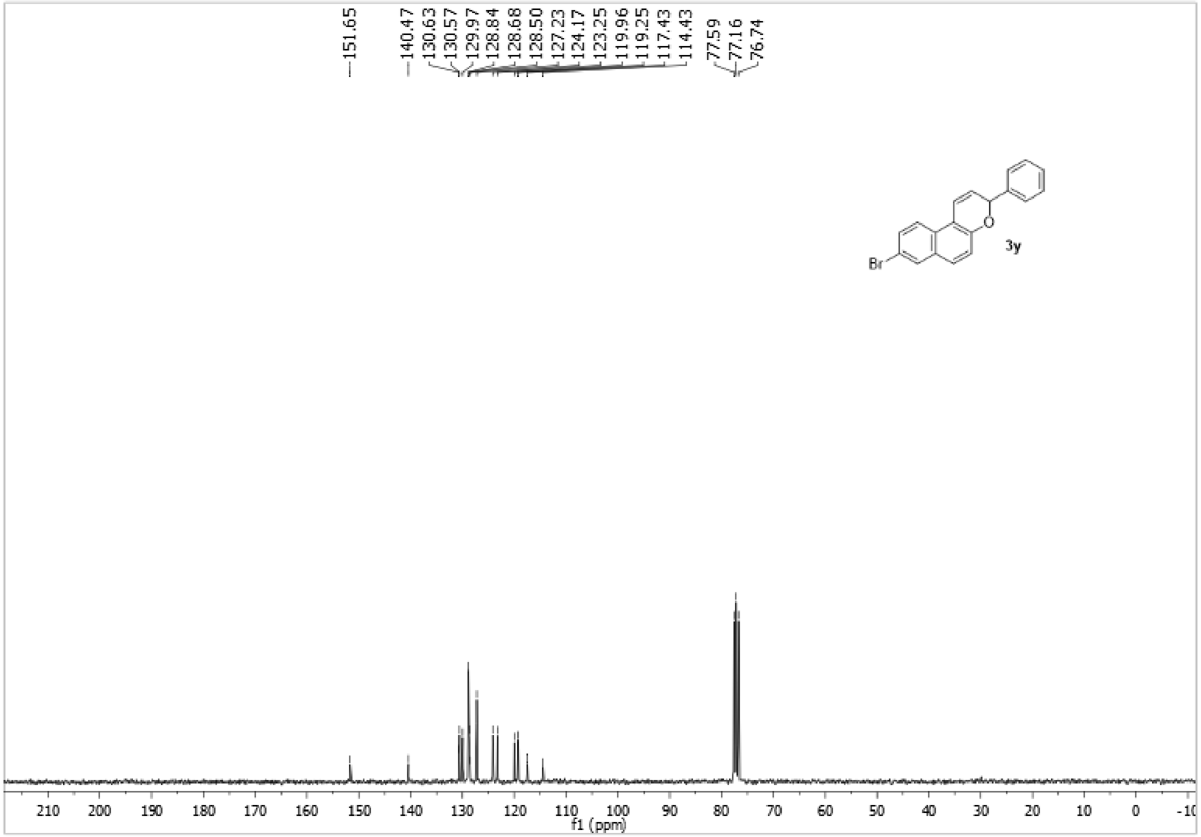
151.65
140.47
130.63
130.57
129.97
128.84
128.68
128.50
127.23
124.17
123.25
119.96
119.25
117.43
114.43
77.59
77.16
76.74
3y
Br
O
f1 (ppm)

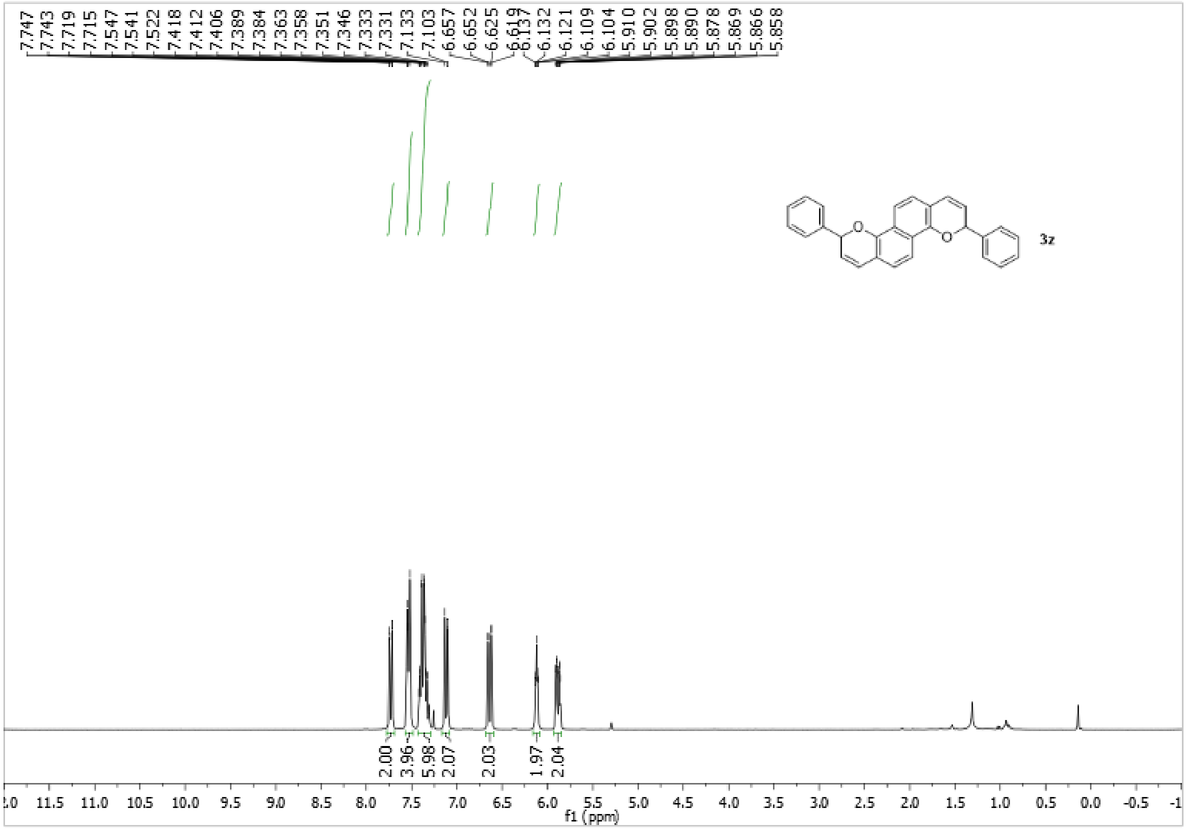

7.747
7.743
7.719
7.715
7.547
7.541
7.522
7.418
7.412
7.406
7.389
7.384
7.363
7.358
7.351
7.346
7.333
7.331
7.133
7.103
6.657
6.652
6.625
6.619
6.137
6.132
6.121
6.109
6.104
5.910
5.902
5.898
5.890
5.878
5.869
5.866
5.858
O
O
3z
2.00
3.96
5.98
2.07
2.03
1.97
2.04
11.5 11.0 10.5 10.0 9.5 9.0 8.5 8.0 7.5 7.0 6.5 6.0 5.5 5.0 4.5 4.0 3.5 3.0 2.5 2.0 1.5 1.0 0.5 0.0 -0.5
f1 (ppm)

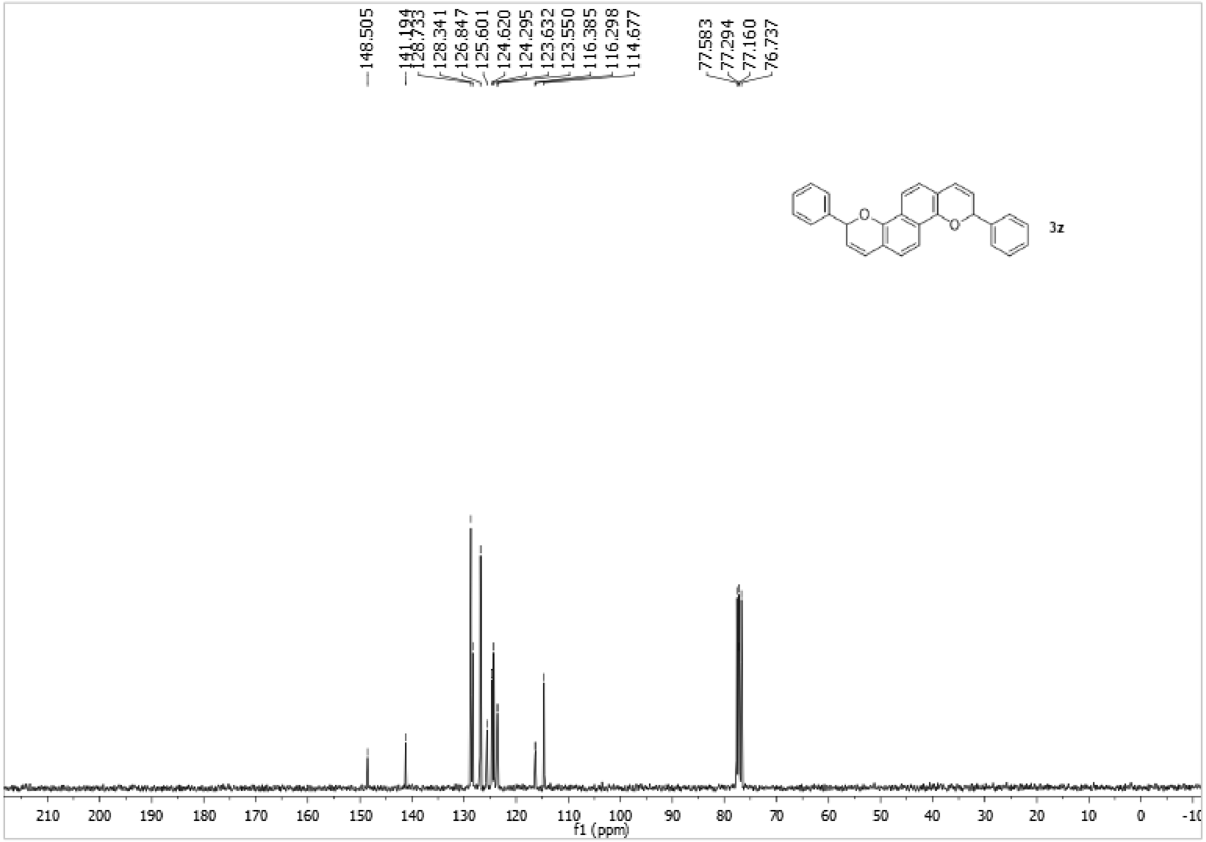

148.505
141.194
128.733
128.341
126.847
125.601
124.620
124.295
123.632
123.550
116.385
116.298
114.677
77.583
77.294
77.160
76.737
O
O
3z
210 200 190 180 170 160 150 140 130 120 110 100 90 80 70 60 50 40 30 20 10 0
f1 (ppm)

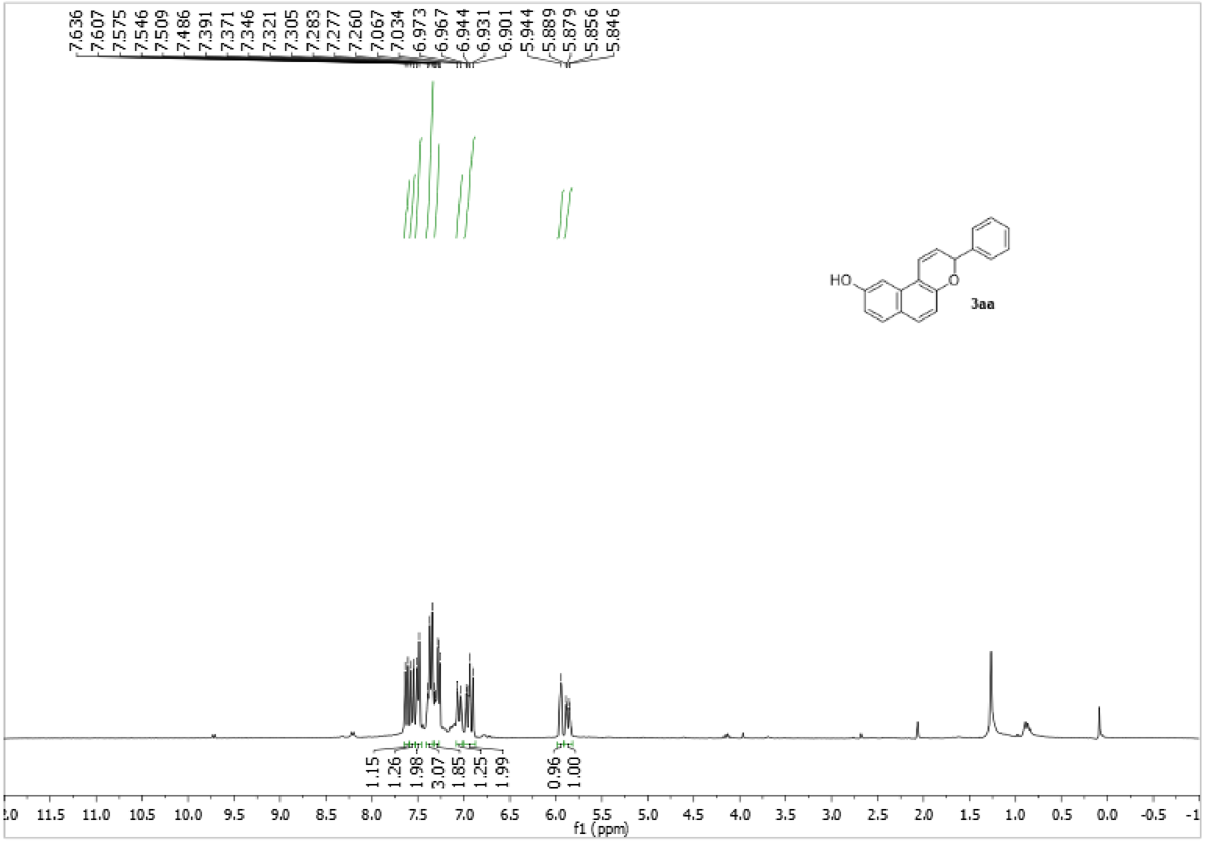
7.636 7.607 7.575 7.546 7.509 7.486 7.391 7.371 7.346 7.321 7.305 7.283 7.277 7.260 7.067 7.034 6.973 6.967 6.944 6.931 6.901 5.944 5.889 5.879 5.856 5.846
HO
O
3aa
1.15 1.26 1.98 3.07 1.85 1.25 1.99 0.96 1.00
f1 (ppm)

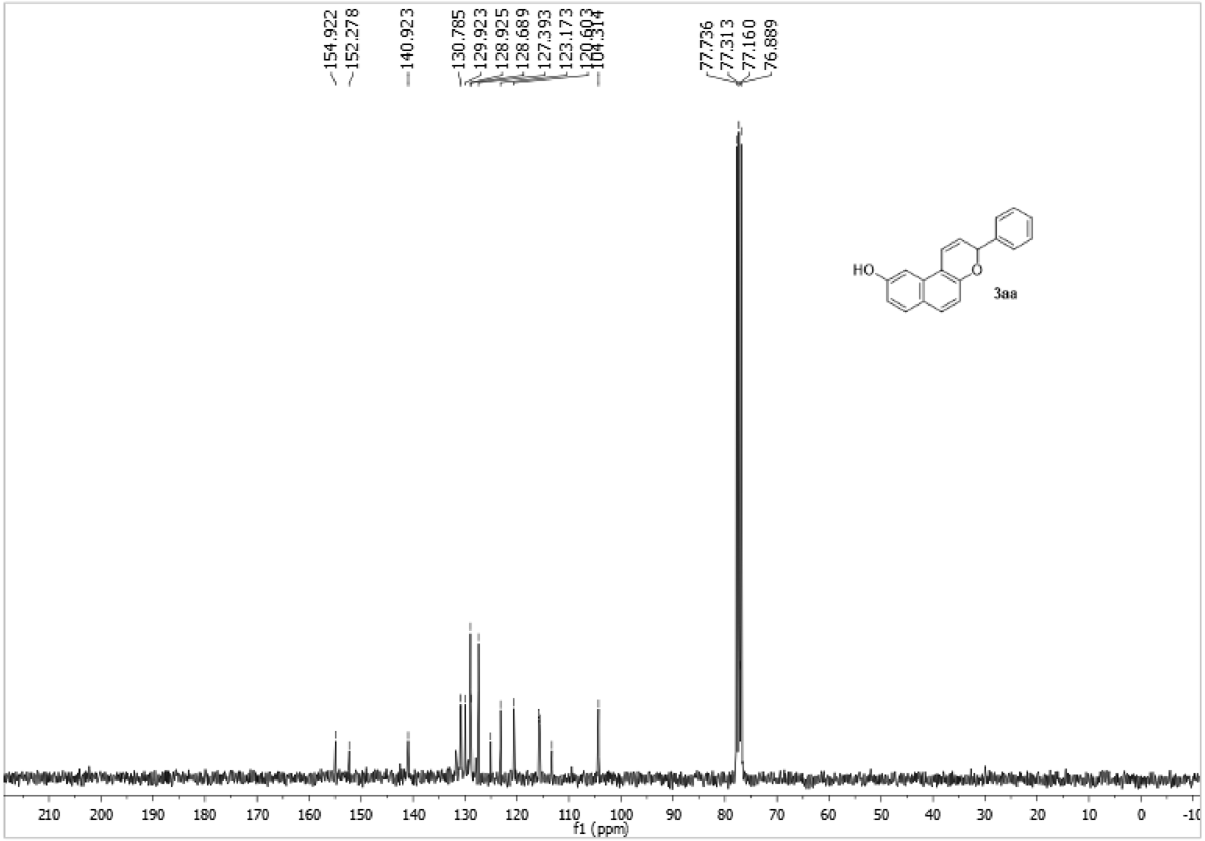
154.922 152.278 140.923 130.785 129.923 128.925 128.689 127.393 123.173 120.603 104.314 77.736 77.313 77.160 76.889
HO
O
3aa
f1 (ppm)

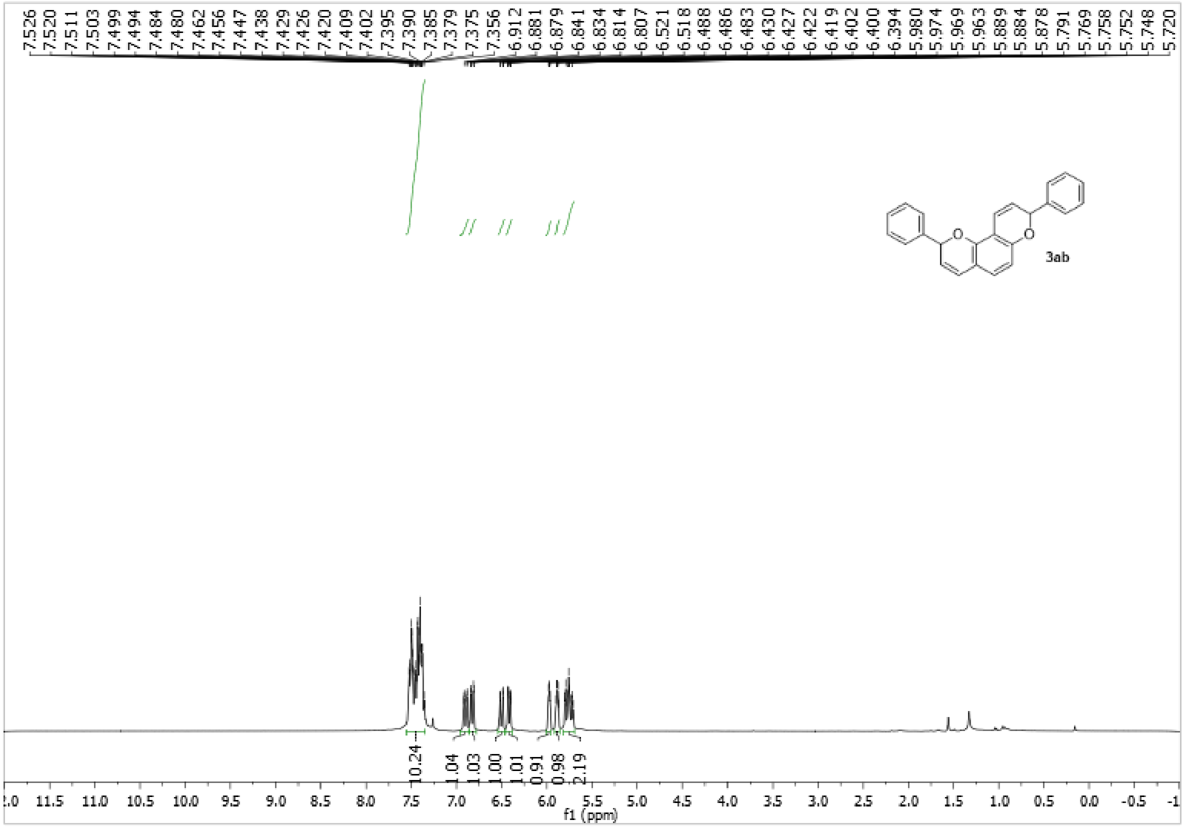
7.526 7.520 7.511 7.503 7.499 7.494 7.484 7.480 7.462 7.456 7.447 7.438 7.429 7.426 7.420 7.409 7.402 7.395 7.390 7.385 7.379 7.375 7.356 6.912 6.881 6.879 6.841 6.834 6.814 6.807 6.521 6.518 6.488 6.486 6.483 6.430 6.427 6.422 6.419 6.402 6.400 6.394 5.980 5.974 5.969 5.963 5.889 5.884 5.878 5.791 5.769 5.758 5.752 5.748 5.720
3ab
10.24
1.04
1.03
1.00
1.01
0.91
0.98
2.19
f1 (ppm)

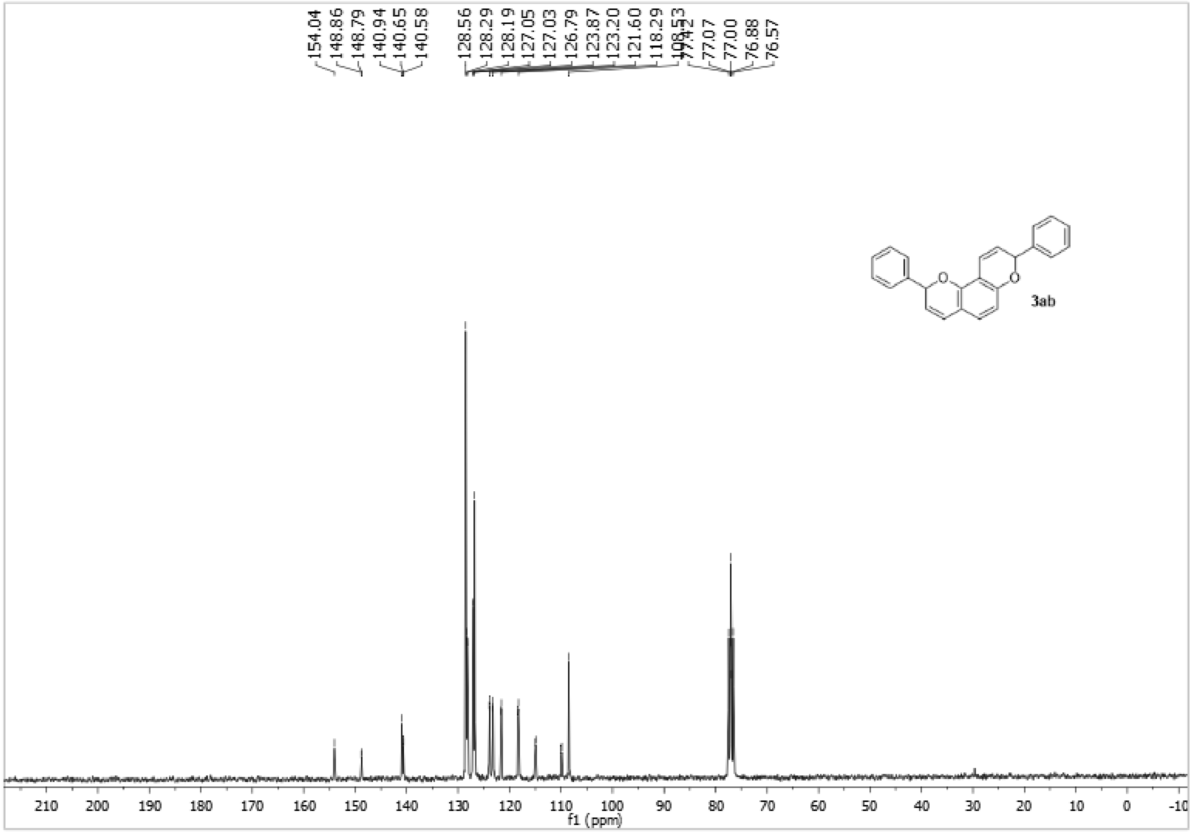
154.04 148.86 148.79 140.94 140.65 140.58 128.56 128.29 128.19 127.05 127.03 126.79 123.87 123.20 121.60 118.29 108.53 77.12 77.07 77.00 76.88 76.57
3ab
f1 (ppm)

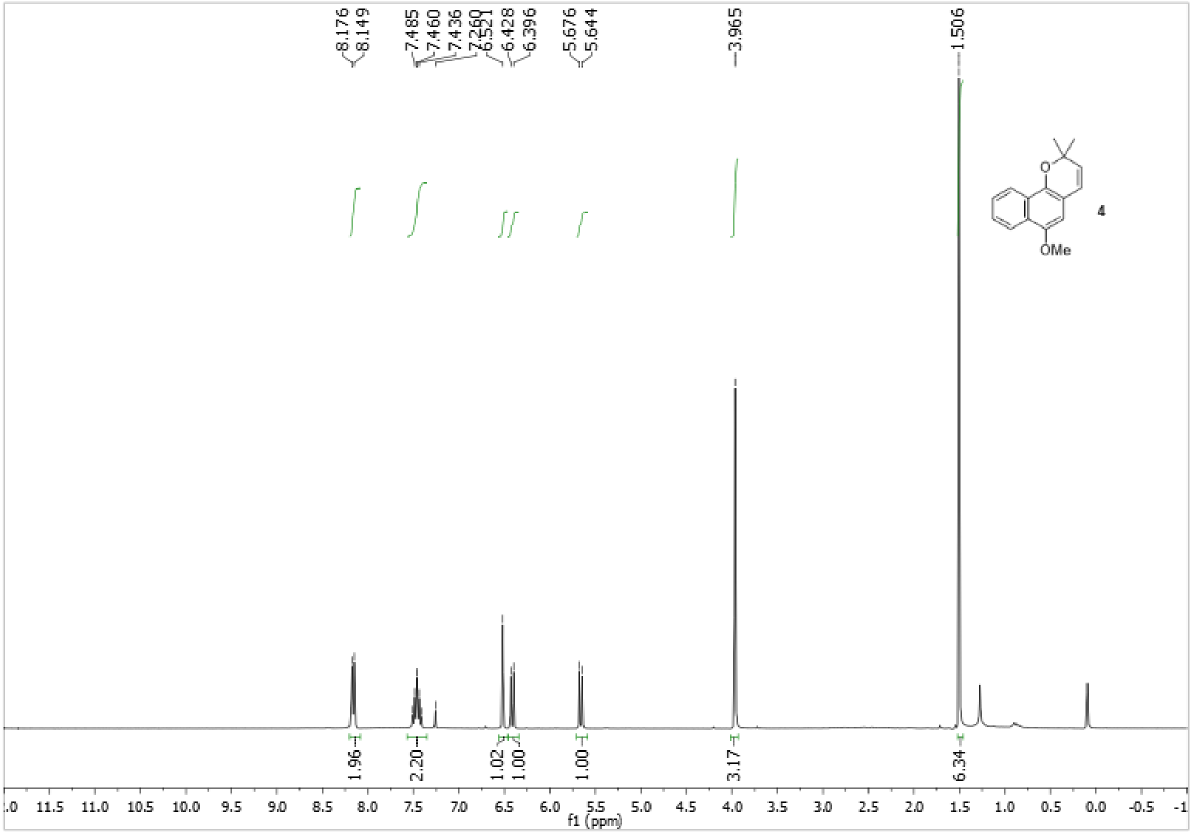
8.176
8.149
7.485
7.460
7.436
7.260
6.521
6.428
6.396
5.676
5.644
3.965
1.506
O
OMe
4
1.96
2.20
1.02
1.00
1.00
3.17
6.34
11.5 11.0 10.5 10.0 9.5 9.0 8.5 8.0 7.5 7.0 6.5 6.0 5.5 5.0 4.5 4.0 3.5 3.0 2.5 2.0 1.5 1.0 0.5 0.0 -0.5
f1 (ppm)

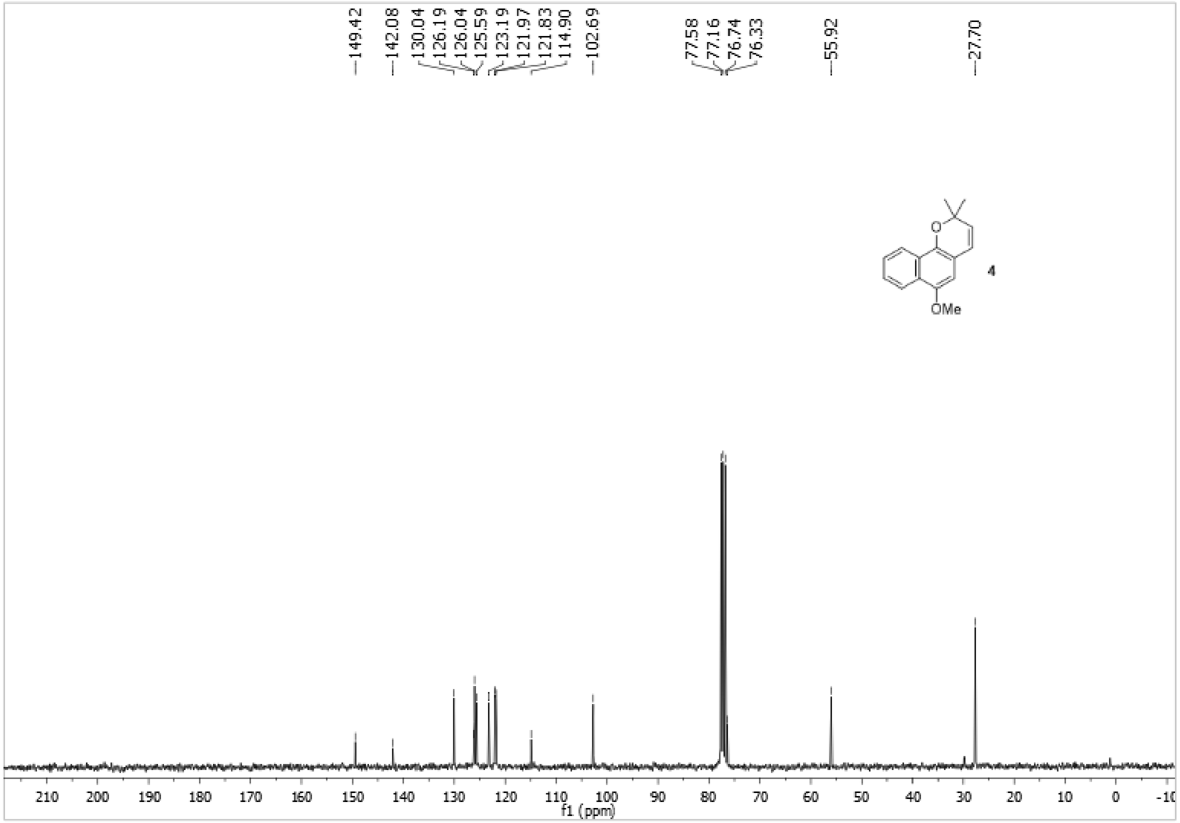
149.42
142.08
130.04
126.19
126.04
125.59
123.19
121.97
121.83
114.90
102.69
77.58
77.16
76.74
76.33
55.92
27.70
O
OMe
4
210 200 190 180 170 160 150 140 130 120 110 100 90 80 70 60 50 40 30 20 10 0
f1 (ppm)
